# MDA5 Plays a Crucial Role in Enterovirus 71 RNA-Mediated IRF3 Activation

**DOI:** 10.1371/journal.pone.0063431

**Published:** 2013-05-01

**Authors:** Rei-Lin Kuo, Li-Ting Kao, Sue-Jane Lin, Robert Yung-Liang Wang, Shin-Ru Shih

**Affiliations:** 1 Research Center for Emerging Viral Infections, College of Medicine, Chang Gung University, Gueishan, Tao-Yuan, Taiwan; 2 Department of Biotechnology and Laboratory Science, College of Medicine, Chang Gung University, Gueishan, Tao-Yuan, Taiwan; 3 Department of Biomedical Sciences, College of Medicine, Chang Gung University, Gueishan, Tao-Yuan, Taiwan; University of Hong Kong, Hong Kong

## Abstract

Induction of type-I interferons (IFNs), IFN-α/β, is crucial to innate immunity against RNA virus infection. Cytoplasmic retinoic acid-inducible gene I (RIG-I)-like receptors, including RIG-I and melanoma differentiation-associated gene 5 (MDA5), are critical pathogen sensors for activation of type-I IFN expression in response to RNA virus infection. MDA5 is required for type-I IFN expression in mouse models in response to infection by picornaviruses, such as encephalomyocarditis virus (EMCV) and coxsackievirus B3. Enterovirus 71 (EV71) belongs to picornaviridae and contains positive-stranded RNA genome that is linked with VPg protein at the 5′ end. Although a recent study showed that EV71 3C protease could suppress RIG-I-mediated IFN-β response, the cytoplasmic RIG-I-like receptor that is directly involved in the recognition of EV71 RNA remains unclear. Using EV71-derived RNA as an agonist, we demonstrate that MDA5 is involved in EV71 RNA-mediated IRF3 activation and IFN-β transcription. Our data also show that overexpression of the MDA5 protein reverses the suppression of IRF3 activation caused by EV71 infection. These results indicate that MDA5 is an important factor for EV71 RNA-activated type-I IFN expression. Furthermore, we also show that EV71 infection enhances MDA5 degradation and that the degradation could be inhibited by a broad spectrum caspase inhibitor.

## Introduction

Enterovirus 71 (EV71) is the causative agent of hand-foot-and-mouth disease which is mild illness in children, but occasionally EV71 infection can result in severe neurological complications, such as encephalitis, aseptic meningitis, and poliomyelitis-like paralysis [1]. Since EV71 was first isolated in California in 1969 [2], many outbreaks with fetal cases have been reported [3]. EV71 belongs to picornaviridae. The virion possesses a single positive-stranded RNA genome that is covalently linked to the virus-encoded VPg protein at the 5′ terminus [4]. The RNA genome consists of a long open reading frame, 5′ and 3′ untranslated regions (UTRs), and a poly(A) tail [5]. The 5′ UTR of EV71 contains a highly ordered secondary structure known as an internal ribosomal entry site (IRES) that directs a cap-independent translation [6]. A single polyprotein is translated from the open reading frame in the EV71 genomic RNA, and functional viral proteins are generated through cleavage of the polyprotein by viral encoded proteases 2A (2A^pro^) and 3C (3C^pro^). EV71 proteases also cleave certain cellular factors. For example, 2A^pro^ cleaves the eukaryotic translation initiation factor 4G (eIF4G), blocking cap-dependent translation in a manner similar to that of the 2A^pro^ of the poliovirus [6]–[8]. In addition, the polyadenylation factor CstF-64 was identified as a substrate for the EV71 3C^pro^. The cleavage of CstF-64 may result in the inhibition of mRNA maturation in host cell [9]. Moreover, these proteases play a key role in EV71-induced apoptosis in infected cells [7], [10].

Type-I IFNs, IFN-α/β, play important roles to initiate cellular antiviral response by activating the genes that establish the target cells to an antiviral state [11]. Certain compositions of viral products, known as pathogen-associated molecular patterns (PAMPs), must be present to initiate the signal cascades that activate type-I IFN promoter in infected cells. Several cellular proteins, such as endosomal Toll-like receptors (TLRs) and cytoplasmic retinoic acid-inducible gene I (RIG-I) like receptors, play the role as pathogen recognition receptors (PRRs) that interact with PAMPs. The melanoma differentiation-associated gene 5 (MDA5) and RIG-I, both of which are cytosolic RIG-I like receptors, have been identified as intracellular PRRs for RNA virus to stimulate type-I IFN expression [12]–[15]. Both the RIG-I and MDA5 proteins consist of a DExD/H-box helicase domain with ATPase activity and two N-terminal caspase activation and recruitment domains (CARDs) that transduce the signal to downstream molecules [16]. After the PRRs bind to the PAMPs, the CARDs are exposed and interact with the adaptor protein named interferon promoter-stimulating factor-1 (IPS-1), also named as Cardif, MAVS, and VISA, on outer mitochondrial membrane [17]–[20]. The formation of this complex then cascades the signal through tumor necrosis factor receptor-associated factors, TANK-binding protein kinase 1 and IKKε, resulting in the phosphorylation of interferon regulatory factor 3 (IRF3). The phosphorylated IRF3 forms a dimer, and translocates to nucleus to activate type-I IFN promoter [21], [22]. LGP2 is an RLR that lacks a CARD domain, and was originally identified as a negative regulator for type-I IFN production [23], [24]. However, the exact role of LGP2 in the intracellular regulation of the innate response remains unclear [25].

Several studies have shown that RIG-I binds to double-stranded RNA (dsRNA) or single-stranded RNA (ssRNA) bearing a 5′ triphosphate [13], [26]–[28], and MDA5 recognizes long dsRNA or highly-ordered RNA structures containing ssRNA and dsRNA [29], [30]. However, the particular sequences or structures within the various viral RNAs may also be recognized by RLRs [31], [32]. Gene knock-out studies have distinguished the roles of RIG-I and MDA5 in response to RNA viruses, revealing RIG-I recognizes paramyxoviruses, othomyxoviruses, and flaviviruses, whereas MDA5 is involved in picornaviruses recognition [14], [33]–[37]. In addition, West Nile virus, Dengue virus, and reoviruses, may be recognized by both of RIG-I and MDA5 [35], [38].

Although Type-I IFNs provide the first line of defense to limit virus replication, many viruses have evolved strategies to inhibit the IFN activation pathways. In picornaviruses, several mechanisms have been identified that suppress RLR signaling, including the degradation of the cytoplasmic RLR signal molecules, such as MDA5 and IPS-1 [39]–[41]. A recent study showed that EV71 3C^pro^ interacts with RIG-I, subsequently disrupting RIG-I-IPS-1 complex that is required for RIG-I-dependent type-I IFN response [42]. This result suggests that RIG-I pathway may be involved in the recognition of EV71, despite EV71 being a picornavirus.

In our current study, we used RNAs derived from EV71-infected cells as agonists to investigate the role of RLRs in the EV71 RNA-mediated induction of type-I IFN response. Based on evidence of IRF3 activation and IFN-β mRNA synthesis, we demonstrate that overexpression of MDA5 or RIG-I protein could enhance the activation of IFN-β transcription in response to EV71 RNA. We also show that knockdown of MDA5, but not RIG-I, expression reduces EV71 RNA-mediated IRF3 activation and IFN-β mRNA production, and that exogenous MDA5 reverses the suppression of IRF3 activation and IFN-β expression caused by EV71 infection. These findings suggest that MDA5 plays a crucial role in innate response to EV71 infection. Furthermore, we show that EV71 infection induces the cleavage of MDA5, but not RIG-I, which may contribute partly to the suppression of type-I IFN production during EV71 infection.

## Materials and Methods

### Cell lines, viruses, and virus infection

HeLa (kindly provided by Dr. Szu-Hao Kung at National Yang-Ming University, Taiwan), Vero (ATCC, CCL81), and RD (ATCC, CCL-136) cell lines were cultured in Dulbecco's modified Eagle medium (DMEM, Gibco, USA) with 10% fetal bovine serum (FBS, Caisson Labs, USA) and 1% penicillin-streptomycin-L-glutamate (PSG) mixture (Gibco, USA). The EV71 strains, TW2231/98 (obtained from the Clinical Virology Laboratory, Department of Pathology, Chang Gung Memorial Hospital, Linkou, Taiwan), BrCr (ATCC, VR784), and MP4 (kindly provided by Dr. Jen-Ren Wang at National Cheng-Kung University) were amplified in Vero cells that were cultured in DMEM containing 2% FBS. The amplified viruses were titrated by plaque formation assay with RD cells. For virus infection, cells were seeded and cultured overnight in 6-well plates. After the indicated treatments below, viruses were added to the treated cells at the indicated multiplicity of infection (MOI). After 1 h adsorption, the infected cells were maintained in DMEM with 2% FBS and 1% PSG. To block EV71 induced apoptosis, a broad spectrum caspase inhibitor, Q-VD-OPH (MP Biomedicals, USA) was added to cells during virus infection at the final concentration 20 nM. MTT (3-[4,5-dimethylthiazol-2-yl]-2,5-diphenyltetrazolium bromide; thiazolyl blue) (Sigma-Aldrich, USA) assay was performed to measure the cell viability after the caspase inhibitor treatment. In brief, after the treatment, the cells were washed with PBS, and then incubated with 1 mg/ml MTT in serum-free medium at 37°C for 3 h. At the end of MTT incubation, the converted dye was dissolved with 0.04N HCl in isopropanol. The absorbance of converted dye was calculated by the absorbance at wavelength 570 nm with subtraction at 630 nm.

### Overexpression and knockdown experiments for MDA5 and RIG-I

N-terminal FLAG-tagged MDA5 and RIG-I expression vectors were kindly provided by Dr. Michael Gale Jr. [23]. Plasmid transfection was performed using the TransIT-LT1 transfection reagent (Mirus Bio, USA) according to the manufacturer's instructions. The lysates of transfected cells were collected at the indicated times for further analysis. Small interfering RNA (siRNA) of MDA5 and RIG-I were designed and synthesized by Sigma-Aldrich Co. To knock down the expression of MDA5 or RIG-I, (MDA5 siRNA sequence: 5′-GUUAUAGUUCUUGUCAAUA & 5′-UAUUGACAAGAACUAUAAC, final concentration: 30 nM; RIG-I siRNA sequence: 5′-GACUAGUAAUGCUGGUGUA & 5′-UACACCAGCAUUACUAGUC, final concentration: 50 nM) was transfected into HeLa or RD cells using the Lipofectamine 2000 transfection reagent (Invitrogen, USA) for the indicated durations.

### EV71 viral RNA preparation and transfection

Total RNA from infected Vero cells was isolated using the Trizol reagent (Invitrogen, USA). To isolate the EV71 genomic RNA, cultured medium from EV71-infected Vero cells was centrifuged at 2000× *g* for 20 min to remove the cells. After measuring the virus titer in the supernatant, viral RNA was extracted using the Trizol reagent. RT-PCR was performed to confirm the existence of EV71 genomic RNA using primers specific to sequences in the 3C region of the EV71 genome. The indicated amounts of EV71 RNA or cellular RNA were transfected into HeLa cells using the Lipofectamine 2000 transfection reagent. After the indicated times and treatments, the cell lysates and RNA were harvested for further analysis.

### Immunoblot analysis

Cells were lysed with 100 mM Tris pH 7.5, 250 mM NaCl, 0.5% sodium deoxycholate, 0.5% NP-40, 1 mM PMSF, and phosphatase inhibitor (Sigma-Aldrich, USA) for the indicated durations. After vortexing and incubating on ice for 10 min, cell lysates were centrifuged at 10,000× *g* for 5 min. Proteins in the lysates were separated by SDS-polyacrylamide gel electrophoresis (SDS-PAGE). The separated proteins were transferred to PVDF membranes, and probed with the anti-EV71 (Millipore, catalogue number: MAB979, USA), anti-EV71 3C [9], anti-total IRF3 (Santa Cruz Biotechnology, USA), anti-phospho-IRF3 (Ser396; Cell Signaling, USA), anti-FLAG M2 (Sigma-Aldrich, USA), anti-MDA5 (Enzo Life Sciences, USA), anti-PARP (Santa Cruz Biotechnology, USA), anti-RIG-I (D14G6; Cell Signaling, USA), or anti-β-actin (Sigma-Aldrich, USA) primary antibodies. After incubating with an HRP-conjugated secondary antibody (GE Healthcare, USA), the specific proteins were visualized using a chemiluminescent HRP substrate (Millipore, USA).

### Analysis for mRNA expression by regular and quantitative RT-PCR

Cellular total RNA was isolated from treated HeLa and RD cells at the indicated time points using the Trizol reagent. Complementary DNA (cDNA) was generated from 2 µg of the RNA by reverse transcription with oligo(dT) primer. To detect the mRNA expression of MDA5, RIG-I, IFN-β and β-actin by regular RT-PCR, the cDNA that was described above was amplified using Taq DNA polymerase and a primer set complementary to the MDA5 gene coding region (forward 5′-TGCATCACGTCAATATGACC-3′; reverse 5′-CCTCATCACTAAATAAACAGC-3′), RIG-I gene coding region (forward 5′-GACCACATCCCAAGCCAAAG-3′; reverse 5′-TCATTTGGACATTTCTGCTG-3′), IFN-β gene coding region (forward 5′-AGAAGGAGGACGCCGCATTG-3′; reverse 5′-TCAGTTTCGGAGGTAACCTG-3′) and primers complementary to the β-actin coding sequence (forward 5′- CTACAATGAGCTGCGTGTGG-3′; reverse 5′-GCTCATTGCCAATGGTGATG-3′). The amplified DNA products were analyzed by agarose gel electrophoresis. The TaqMan gene expression assay and the Applied Biosystems detector were used to quantify the relative amounts of IFN-β mRNA, as previously described [43]. The mRNA of β-actin was used for normalization by the 2^−ΔΔC^_T_ method [44]. Each experiment was performed in triplicate.

## Results

### EV71-derived RNAs induce IRF3 activation and IFN-β expression

**Figure 1:**
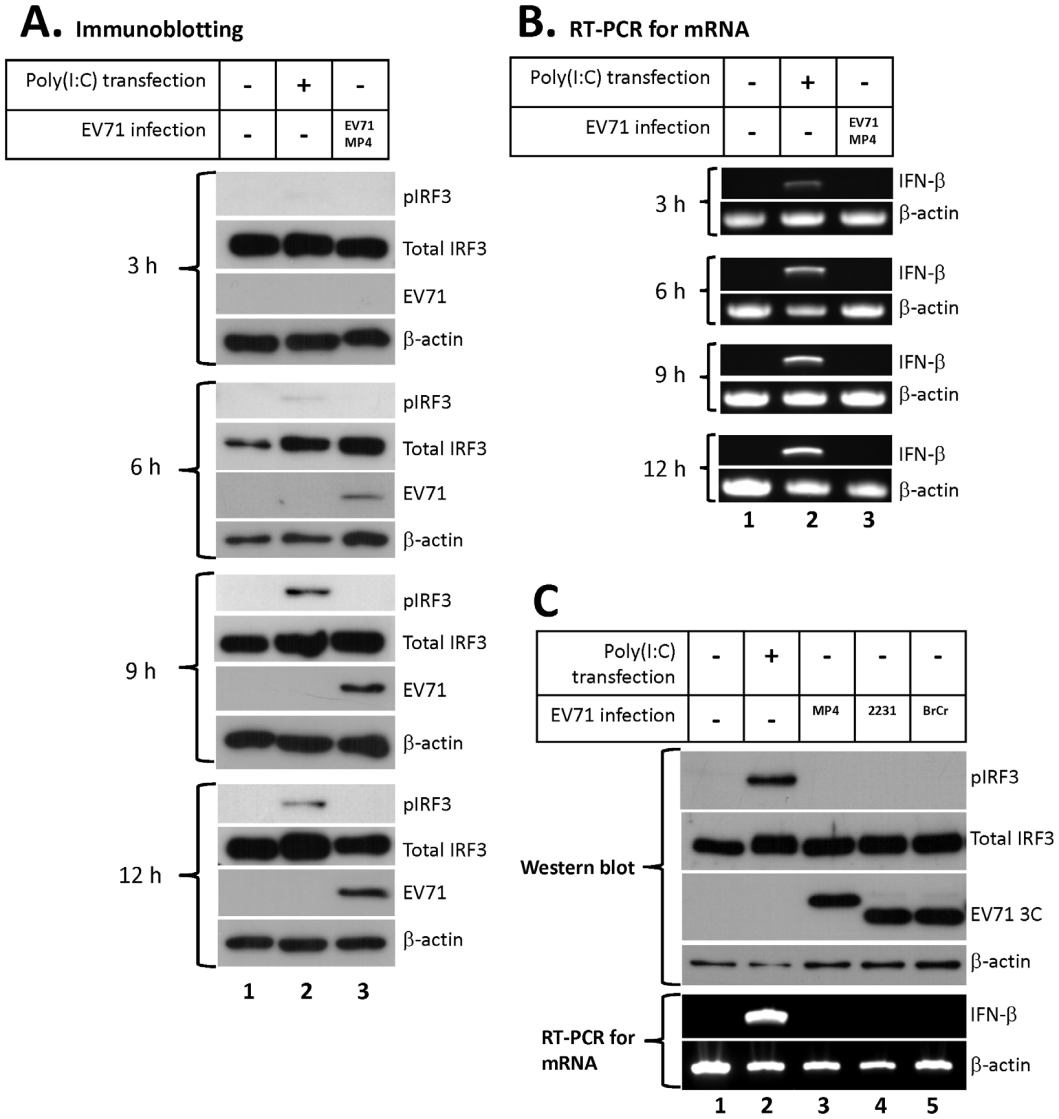
IRF3 is not activated during EV71 infection. HeLa cells were transfected with 10 µg of poly(I:C) or infected with MP4 strain of EV71 at 2 MOI. Cell extracts and total RNA were collected at 3 h, 6 h, 9 h, and 12 h post-transfection or post-infection. Immunoblotting was performed for detecting the presence of phosphorylated IRF3 (pIRF3), total IRF3, EV71 (by anti-EV71 antibody, MAB979, Millipore), and β-actin in the extracts (A). The expression of IFN-β and β-actin mRNA were analyzed by RT-PCR and agarose gel electrophoresis (B). (C) HeLa cells were transfected with 10 µg of poly(I:C) or infected with the MP4, the TW2231, or the BrCr strain of the EV71 virus at 2 MOI. Cell extracts and total RNA were collected at 12 h post-transfection or post-infection. The extracts were analyzed by immunoblotting using anti-phspho-IRF3 (pIRF3), total IRF3, anti-EV71 3C, and anti-β-actin antibodies. Total RNA was analyzed using RT-PCR and agarose gel electrophoresis to detect IFN-β mRNA expression. (MP4, EV71 MP4 strain; 2231, EV71 TW2231/98 strain; BrCr, EV71 BrCr strain).

To examine the type-I IFN response during EV71 infection, HeLa cells were infected with EV71 MP4 strain at MOI 2. At 3 h, 6 h, 9 h, and 12 h post-infection, cell extracts were collected, and then analyzed IRF3 activation by detecting phosphorylated form of IRF3 using immunoblotting. The total RNA from infected cells was also isolated for detecting IFN-β mRNA expression by RT-PCR. As shown in Figure 1A, in contrast to poly(I:C) transfected cells, EV71 infection did not cause phosphorylation of IRF3 (Lane 2 and 3). Consequently, relative to poly(I:C) transfected cells, we did not detect IFN-β mRNA expression in EV71-infected cells (Figure 1B, lane 2 and 3), which is consistent with a previous report [42]. We further examined the IRF3 activation and IFN-β mRNA expression in HeLa cells that were infected with TW2231/98 or BrCr strain of EV71. At 12 h post-infection, neither phosphorylated IRF3 nor IFN-β mRNA was detected in HeLa cells that were infected with MP4, TW2231/98, or BrCr strain of EV71 (Figure 1C, lane 3–5). The data confirm that EV71 infection was not able to activate type-I IFN response by three different strains of EV71 (MP4, TW2231/98, and BrCr). There are two possible explanations for the suppression of type-I IFN expression: (1) The cells could not respond to the RNA derived from EV71 infection, or (2) EV71 blocked the activation of the type-I IFN promoters, as described in the previous studies.

**Figure 2:**
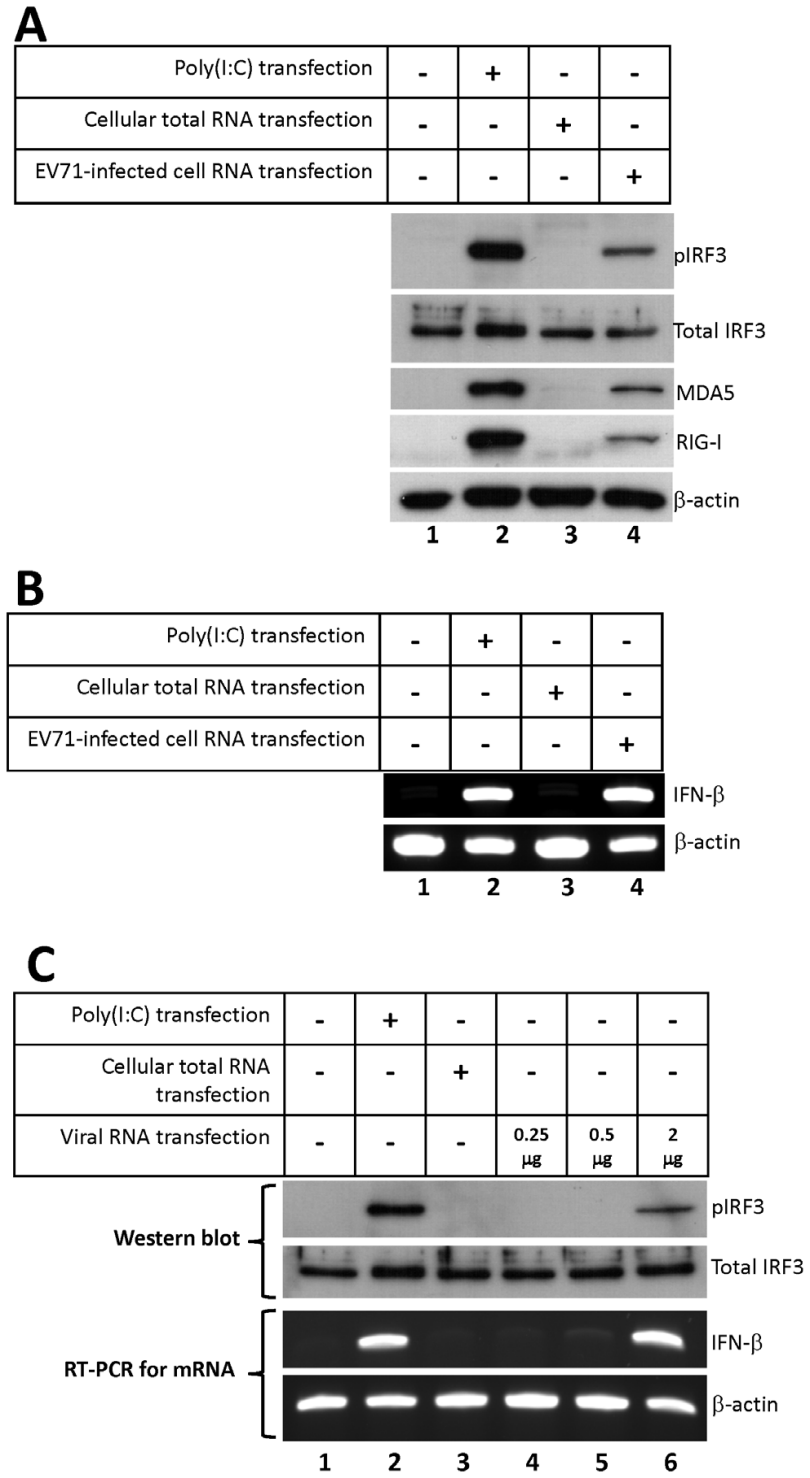
IRF3 is activated by EV71-derived RNA. (A) HeLa cells were transfected with 2 µg of poly(I:C), cellular total RNA of HeLa cells, or total RNA from EV71-infected HeLa cells for 20 h. The activation of IRF3 was analyzed by immunoblotting using anti-phospho-IRF3, anti-MDA5, anti-RIG-I, and anti-β-actin antibodies. Total IRF3 was also detected as a loading control. (B) HeLa cells were treated as described above. The total RNA from the transfected cells was collected and analyzed by RT-PCR for detecting IFN-β mRNA expression. (C) HeLa cells were transfected with 2 µg of poly(I:C), 2 µg of cellular RNA from mock-infected HeLa cells, or the indicated amount of EV71 viral RNA extracted from the supernatant of infected Vero cells for 20 h. The phosphorylated IRF3 and total IRF3 were detected by immunoblotting. Total RNA from transfected cells was collected and IFN-β mRNA expression was evaluated using RT-PCR and agarose gel electrophoresis.

To determine whether the RNAs derived from EV71 infection could induce IRF3 activation, total RNA from EV71/MP4-infected or mock-infected HeLa cells was transfected into HeLa cells. At 20 h post-transfection, phosphorylated IRF3 and two IFN-stimulated cytosolic PRRs, MDA5 and RIG-I, were detected only in the cells that were transfected with the poly(I:C) positive control or the EV71-infected cellular RNA (Figure 2A, lanes 2 and 4). In contrast, the total cellular RNA from mock-infected cells did not induce IRF3 phosphorylation, MDA5, and RIG-I synthesis (Fig. 2A, lane 3). Because transfection of EV71 genomic RNA may result in the production of the virus proteins, we also monitored the expression of the EV71 3C protein in the transfected cells. However, viral 3C protein was not detected at 20 h post-transfection (data not shown). To verify this finding, we repeated the total RNA transfection experiment for detecting the IFN-β mRNA expression in the transfected cells by regular RT-PCR. In contrast to the RNA from mock-infected cells, the total RNA from EV71/MP4-infected HeLa cells could strongly stimulate IFN-β mRNA synthesis (Figure 2B, lane 4).

The total RNA from the EV71-infected cells consisted of genomic RNA, viral replication intermediate RNAs, and cellular RNAs. To rule out the involvement of exogenous cellular RNAs in the observed induction of the type-I IFN expression, EV71/MP4 viral RNA was extracted from virus particles in the supernatant of EV71-infected Vero cells to enrich the EV71 genomic RNA content and eliminate cellular RNA contaminants from the transfection mixture. The enriched EV71 viral RNA was verified by RT-PCR (data not shown), and transfected into HeLa cells. IRF3 phosphorylation and IFN-β mRNA expression were evaluated at 20 h post-transfection. As shown in Figure 2C, phosphorylated IRF3 was detected in the cells transfected with 2 µg of viral RNA (Figure 2C, upper panel, lane 6). The level of IFN-β mRNA was increased in the viral RNA-transfected cells (Figure 2C, lower panel, lanes 5–6). This indicates that the EV71 viral RNA extracted from supernatant of infected cells was sufficient to activate the signaling pathway for type-I IFN expression.

### Overexpression of MDA5 or RIG-I enhances EV71 RNA-mediated induction of type-I IFN expression

**Figure 3:**
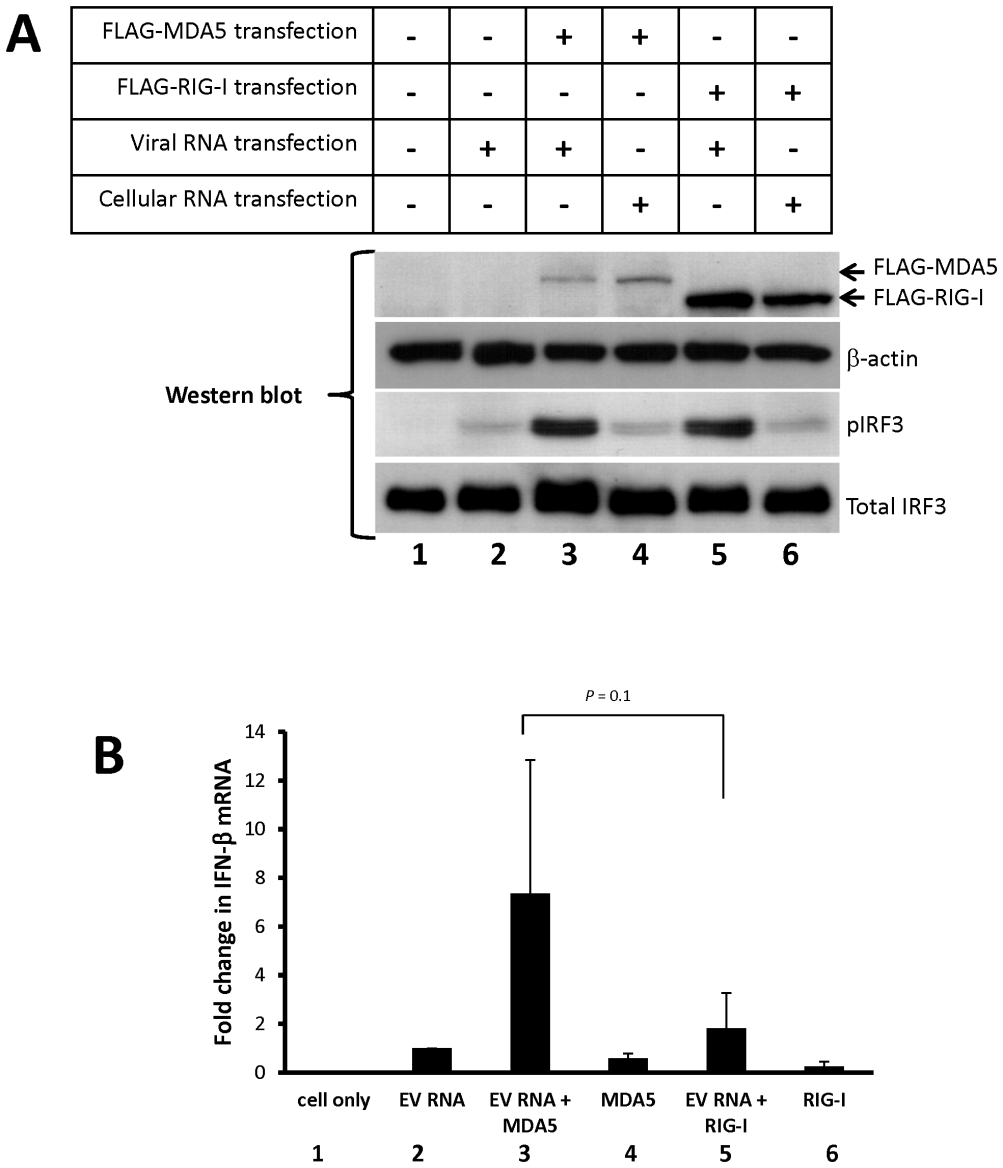
Both MDA5 and RIG-I overexpression enhance the EV71 RNA-mediated IFN-β mRNA expression. (A) HeLa cells were transfected with an empty plasmid or a plasmid expressing the FLAG-MDA5 or FLAG-RIG-I proteins for 24 h. The cells were subsequently transfected with EV71 RNA (1 µg) or total RNA of mock-infected cells (1 µg), and cell extracts and total RNA were collected at 20 h following RNA transfection. The expression of the FLAG-MDA5 and the FLAG-RIG-I proteins was analyzed by immunoblotting using anti-FLAG-M2 primary antibody. IRF3 activation was detected by immunoblotting using anti-phosphorylated IRF3 antibody. RT-PCR and agarose gel electrophoresis were used to detect the IFN-β mRNA. (B) Relative amount of IFN-β mRNA produced in the cells was measured using real-time RT-PCR. The results were calculated by triplicating the experiment described above.

Since the initiation of IFN-β signaling by viral RNAs in the cytoplasm requires the cytosolic receptors for the pathogen-associated molecules, such as the MDA5 and RIG-I proteins, we further investigated the roles of MDA5 and RIG-I in type-I IFN response to presence of EV71 viral RNA. Either MDA5 or RIG-I protein was overexpressed in HeLa cells by transfection with the FLAG-tagged MDA5 or RIG-I expression plasmids. At 24 h post-transfection, the enriched EV71 RNA was transfected into the cells. At 20 h after the EV71 RNA transfection, phosphorylated IRF3 and IFN-β mRNA expression were analyzed by immunoblotting and RT-PCR, respectively. The EV71 RNA alone was sufficient to stimulate IRF3 activation and IFN-β mRNA synthesis (Figure 3A, lane 2), and overexpression of the MDA5 protein dramatically enhanced EV71 RNA-mediated IRF3 activation and IFN-β mRNA expression (Figure 3A, lane 3). The EV71 RNA-mediated IRF3 activation and IFN-β mRNA expression were also increased in the cells in which RIG-I was overexpressed (Fig. 3A, lane 5). To quantify the effects of MDA5 and RIG-I, we triplicated the experiments described above, and applied quantitative RT-PCR for determining the relative amounts of IFN-β mRNA. As shown in Figure 3B, compared to EV71 RNA transfection alone, overexpression of MDA5 could enhance the EV71 RNA-induced IFN-β mRNA expression for 7 folds (Lane 2 vs. 3). However, overexpression of RIG-I could also increase the EV71 RNA-induced IFN-β mRNA expression for around 2 folds (Figure 3B, lane 2 vs. 5). Although overexpression of MDA5 alone forced the activation of IFN-β transcription (Figure 2B, lane 4), the synergistic effect of EV71 RNA and MDA5 overexpression increased IFN-β mRNA expression approximately 13-fold to MDA5 overexpression alone (Figure 3B, lane 3 vs. 4). These results suggest that MDA5 and/or RIG-I could play an important role in the EV71 RNA-induced IFN-β expression.

### Knockdown of MDA5, but not RIG-I, reduces EV71 RNA-induced IFN-β expression

**Figure 4:**
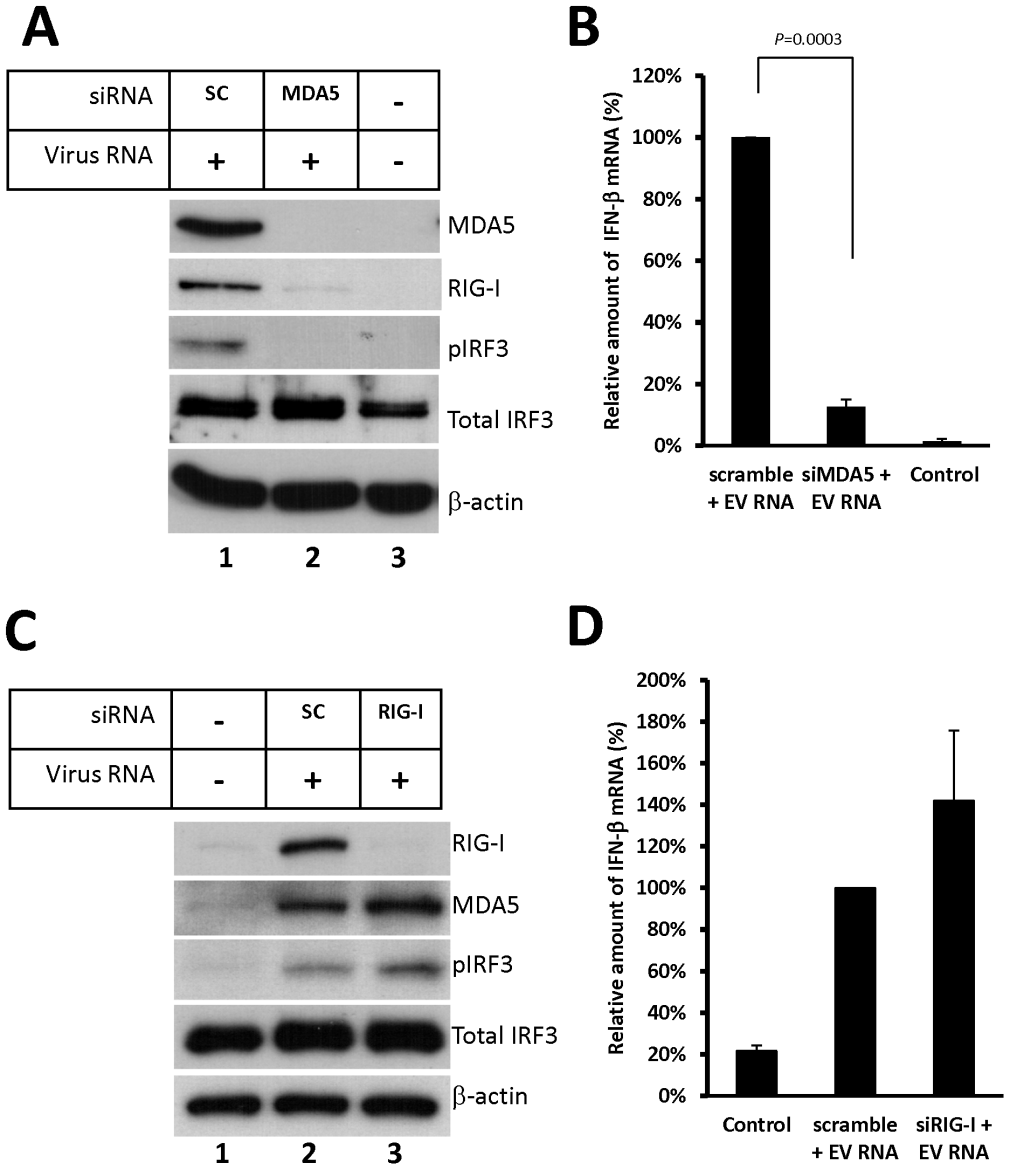
MDA5 plays a crucial role in EV71 RNA-mediated IRF3 activation. (A) HeLa cells were transfected with siRNA against MDA5 or scrambled siRNA for 6 h, followed by transfection with EV71 RNA for 24 h. Extracts from the transfected cells were collected, and the expression of MDA5 and RIG-I was analyzed by immunoblotting using an anti-MDA5 or anti-RIG-I antibody. IRF3 activation was assayed by immunoblotting using anti-phospho-IRF3 and anti-total-IRF3 antibodies. (B) Total RNA was isolated from the transfected HeLa cells. Relative amount of the IFN-β mRNA was measured using real-time RT-PCR. (C) HeLa cells were transfected with scrambled siRNA or siRNA against RIG-I for 24 h, followed by transfection with EV71 RNA for 20 h. Cell extracts and total RNA were collected from the transfected HeLa cells. The expression of RIG-I, MDA5, phosphorylated IRF3, total IRF3, and β-actin was detected by immunoblotting. (D) Relative amount of IFN-β mRNA in RIG-I knockdown cells was measured by real-time RT-PCR.

To verify the involvement of MDA5 and RIG-I in EV71 RNA-mediated type-I IFN response, the induced expression of endogenous MDA5 or RIG-I was knocked down using siRNA in HeLa cells. Initially, HeLa cells were transfected with the MDA5 siRNA, and incubated for 6 h. The EV71 viral RNA was transfected to the MDA5 knockdown cells. As shown in Figure 4A, MDA5 expression was induced by EV71 viral RNA (Lane 1), and MDA5 siRNA reduced MDA5 expression (Lane 2). We further analyzed the activation of IRF3 in both scrambled siRNA- and MDA5 siRNA-treated cells. The results showed that EV71 viral RNA failed to induce IRF3 activation in MDA5 knockdown cells, whereas the scrambled siRNAs did not affect the phosphorylation of IRF3 (Figure 4A, lanes 1–2). To confirm and quantify the effect of MDA5 knockdown on the EV71 viral RNA-induced type-I IFN expression, IFN-β mRNA was quantified using real-time RT-PCR in both control and MDA5 siRNA-treated cells. As shown in Figure 4B, the level of IFN-β mRNA in the MDA5 siRNA-treated cells was decreased to 13% of the scrambled siRNA-treated cells. A similar result was obtained by using RD cells (Figure S1). However, because RIG-I is an IFN-stimulated gene, the reduction of IFN-β could also decrease RIG-I expression in the cells that were co-transfected with EV71 RNA and MDA5 siRNA. As shown in Figure 4A, EV71 RNA-induced RIG-I was also reduced by MDA5 knockdown (Figure 4A, lane 1 vs. 2). Thus, this MDA5 knockdown approach did not completely exclude that RIG-I was involved in EV71 RNA-mediated IFN-β gene activation. To examine the role of endogenous RIG-I in EV71 RNA-mediated IFN-β production, HeLa cells were transfected with the RIG-I siRNA for 24 h, and then transfected with EV71 viral RNA. At 20 h after viral RNA transfection, cell extracts and total RNA of the transfected cells were isolated and analyzed for the expression of RIG-I, MDA5, IFN-β, and β-actin. As shown in Figure 4C, the expression of RIG-I and MDA5 was increased by EV71 viral RNA transfection (Lane 2), and RIG-I siRNA sufficiently reduced the amount of RIG-I, but not MDA5 (Lane 3). Interestingly, after analyzing the phosphorylated IRF3 and IFN-β mRNA, the reduction of RIG-I did not decrease IRF3 activation and IFN-β mRNA expression (Figure 4C, lane 3; Figure 4D). To verify the effect of RIG-I siRNA, an in vitro-transcribed (IVT) RNA was transfected to the RIG-I knockdown cells. The result showed that RIG-I siRNA was sufficient to reduce the IVT RNA-stimulated IRF3 activation and IFN-β mRNA production (Figure S2). These experiments verified that MDA5 is required for EV71 viral RNA-mediated IRF3 activation and IFN-β induction.

### Overexpression of MDA5 or RIG-I reverses the inhibition of IRF3 activation upon EV71 infection

**Figure 5:**
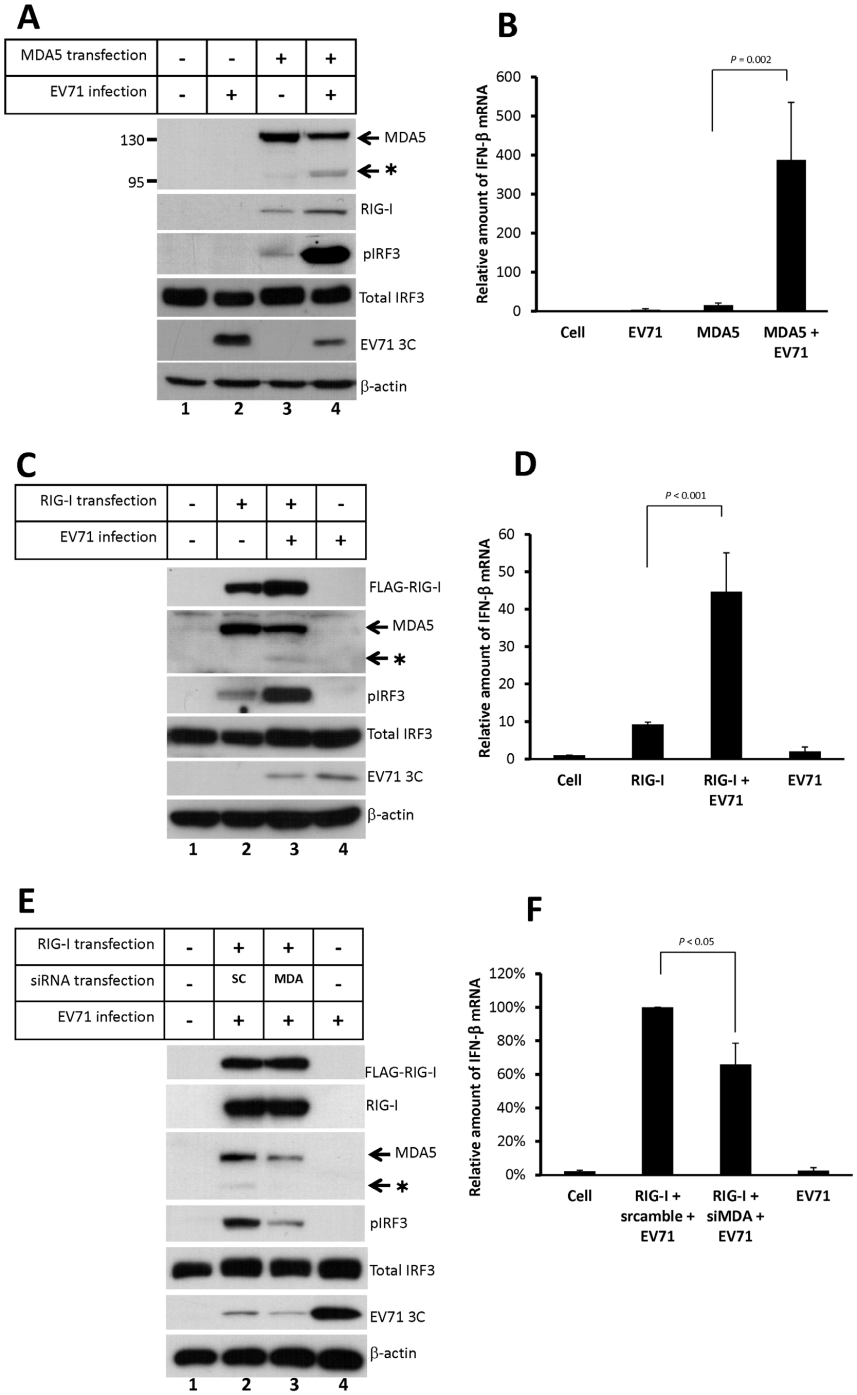
Overexpression of MDA5 or RIG-I reverses the inhibition of IRF3 activation during EV71 infection. HeLa cells were transfected with an empty plasmid or a plasmid expressing the FLAG-MDA5 protein for 36 h, and were subsequently infected with the MP4 strain of the EV71 virus at 2 MOI. At 9 h post-infection, cell extracts were analyzed by immunoblotting using anti-MDA5, anti-RIG-I, anti-phospho-IRF3, anti-total IRF3, anti-EV71 3C, and anti-β-actin antibodies (A). (B) Relative amount of IFN-β mRNA produced in the cells was measured using real-time RT-PCR. (C) HeLa cells were transfected with an empty plasmid or a plasmid expressing the FLAG-RIG-I protein for 36 h, and were subsequently infected with the EV71 at 2 MOI. At 9 h post-infection, cell extracts were analyzed by immunoblotting using anti-FLAG, anti-MDA5, anti-RIG-I, anti-phospho-IRF3, anti-total IRF3, anti-EV71 3C, and anti-β-actin antibodies. The asterisk indicates the putative cleavage product of the MDA5 protein. The relative amount of IFN-β mRNA was quantitated by real-time RT-PCR (D). (E) HeLa cells were transfected with RIG-I expressing plasmid for 24 h, and then transfected with MDA5 siRNA for 18 h. The co-transfected cells were infected with EV71 at MOI 2. At 9 h post-infection, cell extracts were detected the RIG-I, MDA5, phosphorylated IRF3, total IRF3, and β-actin by immunoblotting. Total RNA was also collected for detecting IFN-β mRNA by real-time RT-PCR (F).

We have previously shown that MDA5 is involved in EV71 viral RNA-activated type-I IFN expression. However, IRF3 activation and IFN-β mRNA were not detected upon EV71 infection as shown in Figure 1. To further confirm that MDA5 participates in the EV71 RNA-mediated IRF3 activation upon infection, we evaluated IRF3 phosphorylation in HeLa cells that were overexpressing FLAG-tagged MDA5 protein. At 36 h post-transfection, the cells were infected with EV71 for 9 h (MOI = 2). As shown in Figure 5A, overexpression of MDA5 alone slightly resulted in IRF3 phosphorylation (Lane 3), which is consistent with data from previous studies (Figure 3A, lane 4). In addition, EV71 infection significantly enhanced IRF3 phosphorylation in MDA5-transfected cells (Figure 5A, lane 4), whereas IRF3 phosphorylation was not observed in the cells that were transfected with the empty control plasmid (Figure 5A, lane 2). Consequently, the strong activation of type-I IFN response reduced EV71 replication as shown by viral 3C protein expression (Figure 5A, lane 2 vs. 4). To verify the observation of IRF3 activation, a real-time RT-PCR was performed to quantify relative amount of IFN-β mRNA expression. The result showed that overexpression MDA5 dramatically enhanced IFN-β mRNA level in EV71-infected cells (Figure 5B). These data indicate that MDA5 may participate in activation of type-I IFN expression in response to EV71 infection. However, expression of IFN-β could also stimulate RIG-I expression (Figure 5A, lane 4). Thus, the exact role of RIG-I in EV71-infected cells was further investigated.

To examine the effect of RIG-I overexpression in EV71 infection, a plasmid expressing FLAG-tagged RIG-I was transfected to HeLa cells for 36 h, and then infected with EV71. At 9 h after EV71 infection, cell extracts were collected for immunoblotting analysis. As shown in Figure 5C, IRF3 activation was detected in RIG-I overexpressing cells (Figure 5C, lane 2 and 3), and EV71 infection could increase the level of activated IRF3 (Figure 5C, lane 3). The quantitative RT-PCR showed that RIG-I overexpression could enhance IFN-β mRNA production in EV71-infected cells (Figure 5D). However, overexpression of RIG-I could also induce the expression of endogenous MDA5 (Figure 5C, lane 2 and 3). Thus, the effects of MDA5 and RIG-I in EV71-infected cells were compared in parallel. As shown in Figure S3, although transfection of RIG-I could induce MDA5 expression (Figure S3A, lane 5 and 6) and enhance IFN-β mRNA production in EV71-infected cells, the effect of RIG-I transfection was much less than the effect of MDA5 overexpression (Figure S3B). This result suggested that MDA5 may play more important role in IFN-β production for responding EV71 infection. To confirm this suggestion for MDA5, HeLa cells were transfected with RIG-I expressing plasmid for 24 h, and then transfected with MDA5 siRNA to reduce the induced expression of MDA5. At 18 h after siRNA transfection, the co-transfected cells were infected with EV71 at MOI 2. At 9 h post-infection, cell extracts and total RNA were collected for analyzing IFN-β gene activation. Although siRNA against MDA5 could only suppress MDA5 expression partially, the phosphorylated IRF3 was obviously reduced in the infected cells (Figure 5E, lanes 2 and 3). Consistently, IFN-β mRNA was significantly decreased upon MDA5 siRNA transfection (Figure 5F). These results implied that MDA5 plays a significant role for activating the expression of type-I IFN upon EV71 infection.

### EV71 infection causes MDA5 degradation

**Figure 6:**
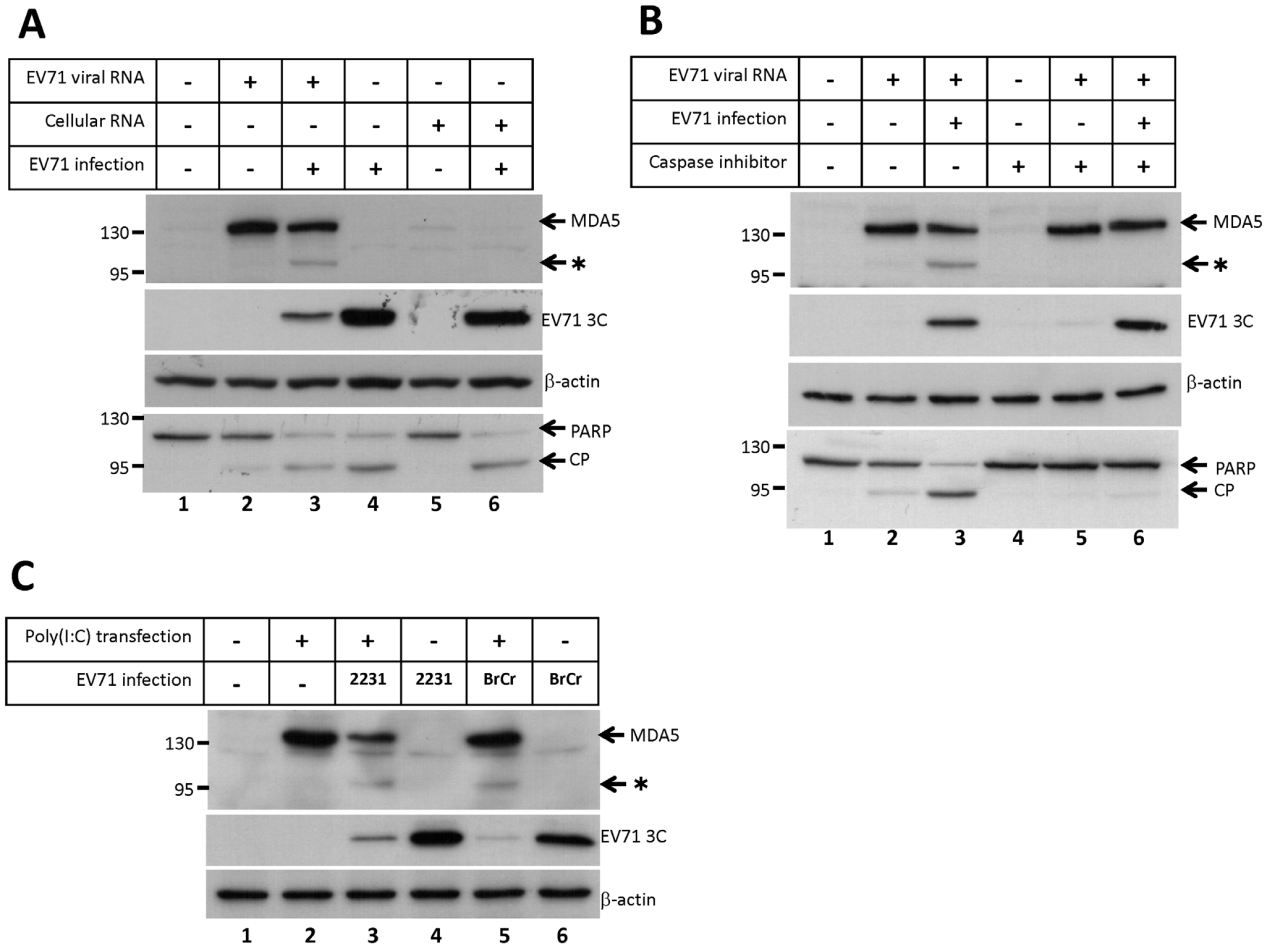
EV71 induces the cleavage of endogenous MDA5 protein. (A) HeLa cells were transfected with 1 µg of EV71 RNA, 1 µg of total RNA from mock-infected cells, or transfection reagent alone. At 14 h post-transfection, the cells were infected with the MP4 strain of the EV71 virus at 2 MOI. At 9 h post-infection, cell extracts were analyzed by immunoblotting using anti-MDA5, anti-EV71 3C, anti-β-actin, and anti-PARP antibodies. (B) HeLa cells were transfected with 1 µg of EV71 RNA for 14 h, followed by EV71 infection. During the virus infection, the cells were treated with a broad spectrum caspase inhibitor, Q-VD-OPH, at final concentration of 20 nM. At 9 h post-infection, cell extracts were collected and analyzed by immunoblotting using anti-MDA5, anti-EV71 3C, anti-β-actin, and anti-PARP antibodies. (C) HeLa cells were transfected with 2 µg of poly(I:C) or transfection reagent alone for 16 h, and subsequently infected with the TW2231/98 or the BrCr strain of the EV71 virus at 2 MOI. At 9 h post-infection, cell extracts were analyzed by immunoblotting using anti-MDA5, anti-EV71 3C, and anti-β-actin antibodies. The asterisk indicates the putative cleavage product of the MDA5 protein, and CP indicates the cleavage product of the PARP protein.

In our evaluation of MDA5 overexpression, we found that EV71 infection resulted in the appearance of a shorter polypeptide (approximately 95 kDa) that was detected in immunoblots using the anti-MDA5 antibody (Figure 5A, lane 4, asterisk), suggesting that MDA5 is cleaved upon EV71 infection. To monitor the effect of EV71 infection on the endogenous MDA5 molecules, HeLa cells were transfected with EV71 viral RNA to induce endogenous MDA5 expression. At 14 h post-transfection, the cells were infected with EV71 (MOI = 2) for 9 h, and the MDA5 protein was analyzed by SDS-PAGE and immunoblotting using the anti-MDA5 antibody. As shown in Figure 6A, the expression of the MDA5 protein in HeLa cells was induced following EV71 viral RNA transfection (Lanes 2 and 3), but was not induced following EV71 infection alone or cellular RNA transfection (Lanes 4–6). Following the EV71 infection of cells that were previously transfected with EV71 RNA, the level of previously-induced full-length MDA5 protein was reduced and a previously-absent 95 kDa protein band was detected (Fig. 6A, lane 3, asterisk). It had been reported that poliovirus infection induces cleavage of the MDA5 protein, whereas infection with echovirus 1 or rhinovirus 16 does not, and that the MDA5 cleavage is concurred with poly(ADP-ribose) polymerase (PARP) cleavage, a hallmark of apoptosis [40]. Because EV71 induces apoptosis and initiates the caspase cascades in infected cells [7], [10], the 95 kDa MDA5 cleavage product generated in the infected cells may be dependent on the caspase activation during EV71 infection. To verify whether EV71-induced caspase cascade could generate the 95 kDa MDA5 polypeptide, we also examined the cleavage of PARP, which is dependent on caspase activation, using immunoblotting with anti-PARP antibody. As shown in Figure 6A, EV71 infection resulted in PARP cleavage (Lanes 3, 4 and 6), which concurred with MDA5 degradation (Lane 3). The experiment was also repeated at 3 h and 6 h post-infection. The result showed that MDA5 cleavage could be detected at 6 h post-infection which coincided with viral protein expression and PARP cleavage (Figure S4). Although the relative amount of viral RNA-induced IFN-β mRNA was changed upon EV71 infection at 6 h post-infection (Figure S4), other approach is required to conclude the effect on IFN-β production (see Discussion). To further correlate the MDA5 cleavage with the caspase cascade initiated by EV71 infection, a broad spectrum caspase inhibitor, Q-VD-OPH, was applied to block caspase cascade during EV71-induced apoptotic processes. As described above, MDA5 was induced by EV71 viral RNA transfection in HeLa cells. At 14 h after EV71 RNA transfection, the cells were infected with EV71 in presence or absence of Q-VD-OPH for 9 h. As shown in Figure 6B, the caspase inhibitor could sufficiently inhibit the cleavage of PARP (Lanes 5 and 6). Under the treatment, the 95 kDa MDA5 degradation product was not detected by immunoblotting (Figure 6B, lane 6). These data suggest that the MDA5 cleavage is caspase-dependent as previous study in poliovirus.

To determine whether the observed EV71-induced MDA5 cleavage was strain-specific, the expression of MDA5 was induced in HeLa cells by transfection with poly(I:C), followed by infection with the TW2231/98 or the BrCr strain of the EV71 virus. We found that both the TW2231/98 and the BrCr strain also induced MDA5 cleavage (Figure 6C, lanes 3 and 5). Because the expression of MDA5 is critical to the recognition of viral RNA and enhances downstream antiviral signaling, the induction of MDA5 by EV71 viral RNA or poly(I:C) may result in suppression of EV71 replication. Immunoblotting analysis of the EV71 3C protein showed that EV71 replication was limited in cells that had been transfected with EV71 RNA or poly(I:C) (Figure 6A, lane 3; Figure 6C, lanes 3 and 5).

Our previous data also showed that overexpression of RIG-I could be also involved in EV71 viral RNA-induced IFN-β expression. To examine whether EV71 infection induced RIG-I cleavage, HeLa cells were shortly transfected with either the FLAG-tagged MDA5 or the FLAG-tagged RIG-I expression plasmid for 24 h, and then infected with EV71 (MOI = 2). At 9 h post-infection, the integrity of the FLAG-tagged proteins was analyzed by immunoblotting with an anti-FLAG antibody. As shown in Figure S5A, the intensity of the full-length FLAG-MDA5 band (Lane 4) was reduced and the MDA5 cleavage product was detected (Figure S5B, lane 3) following EV71 infection. In contrast, the intensity of the RIG-I band was not affected by EV71 infection (Figure S5A, lane 6), indicating that RIG-I cleavage did not occur at this time point.

**Figure 7:**
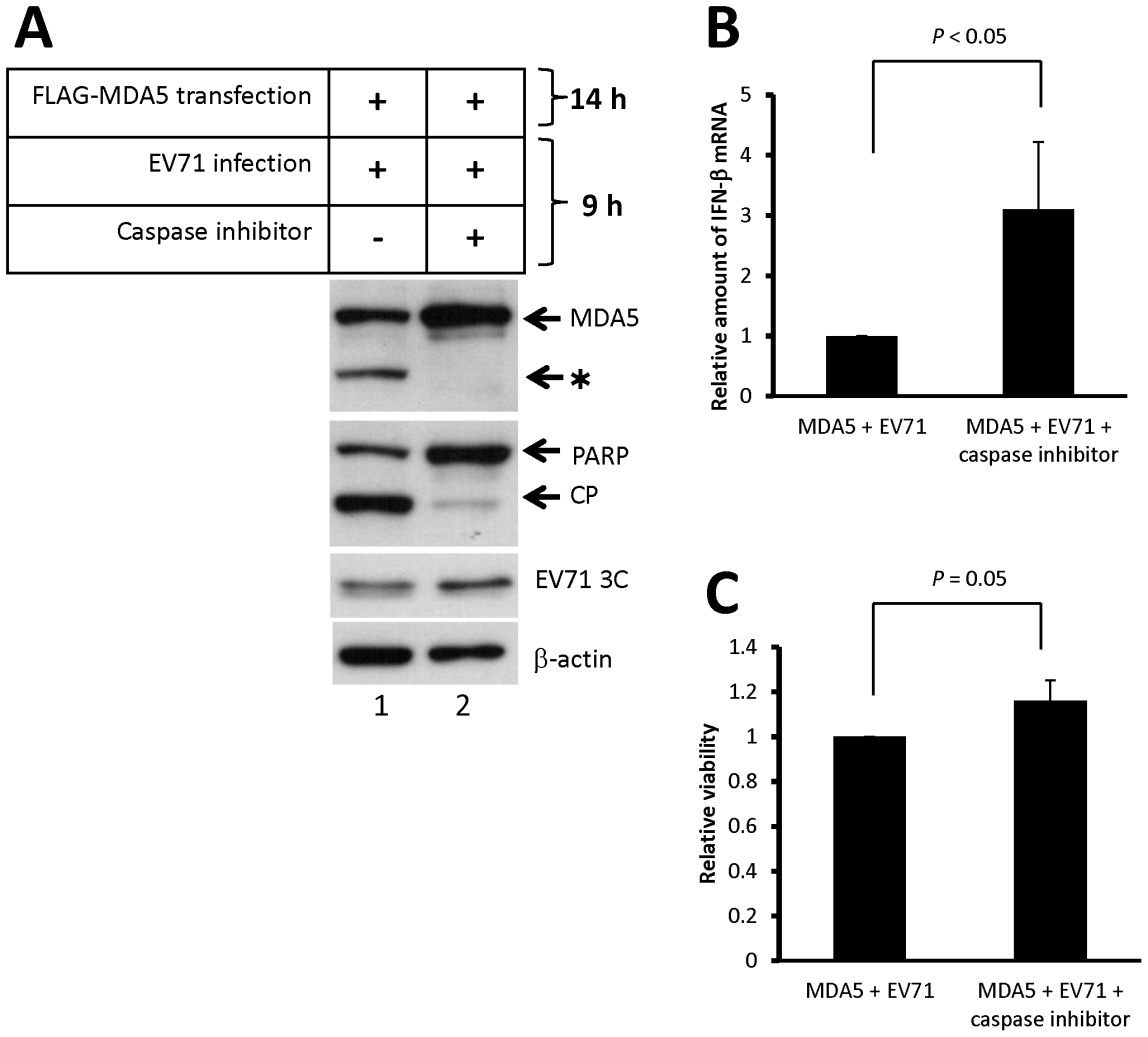
Examining the effect of caspase-dependent MDA5 cleavage on IFN-β mRNA production upon EV71 infection. HeLa cells were transfected with a plasmid expressing the FLAG-MDA5 for 14 h The transfected cells were subsequently infected with EV71/MP4 strain in presence or absence of a broad spectrum caspase inhibitor, Q-VD-OPH, at final concentration of 20 nM. Cell extracts and total RNA were collected at 9 h post-infection. The cell extracts were assayed by immunoblotting using anti-MDA5, anti-PARP, anti-V71 3C, and anti-β-actin antibodies (A). Relative amount of IFN-β mRNA in the cells was measured by real-time RT-PCR (B). (C) MTT assay was performed to determine the relative cell viability.

To determine whether MDA5 cleavage induced by EV71 affects the production of IFN-β mRNA, HeLa cells were transfected with a FLAG-MDA5 expressing plasmid for 14 h, and then infected with EV71 for 9 h in presence or absence of the caspase inhibitor, Q-VD-OPH. As shown in Figure 7A, PARP and MDA5 were cleaved in EV71-infected cells (Lane 1), which is consistent to our previous results. By treating the cells with Q-VD-OPH during EV71 infection, the cleavage of PARP and MDA5 was dramatically reduced (Figure 7A, lane 2). We then measured the relative amount of IFN-β mRNA by real-time RT-PCR. As shown in Figure 7B, the level of IFN-β mRNA was increased by Q-VD-OPH treatment. In spite of the fact that the caspase inhibitor could suppress exogenous MDA5 degradation and enhance IFN-β mRNA production during EV71 infection, the caspase inhibitor could also increase cell viability around 20% upon EV71 infection (Figure 7C) which may also affect IFN-β transcription and EV71 replication. Therefore, the overall effect on the IFN-β production and virus replication remains further determination. Experiments with different time-point design were also performed to confirm the effect on IFN-β production by the caspase inhibitor treatment. HeLa cells were transfected by FLAG-MDA5 for 22 h, and following EV71 infection for 12 h. Similar results were obtained after analyzing the MDA5 cleavage and IFN-β mRNA production (Figure S6A and S6B).

## Discussion

Although EV71 infection does not induce IRF3 activation, we showed that the presence of EV71 viral RNA in the cytoplasm of cells activates IRF3 and induces IFN-β gene expression. Based on these results, we further demonstrated that the overexpression of either MDA5 or RIG-I protein enhances the EV71 viral RNA-mediated induction of IFN-β expression. We also showed that the diminished MDA5, but not RIG-I, expression was correlated with the reduction of EV71 RNA-mediated IRF3 activation in gene knockdown experiments. Furthermore, the suppression of IRF3 activation by EV71 infection was strongly reversed by the pre-existing MDA5 protein. These data indicate that the MDA5 protein plays a crucial role in the activation of IRF3 and the induction of IFN-β transcription in response to EV71. Although the EV71 3C protein has been reported to down-regulate IFN-β transcription through interactions with host RIG-I protein or cleavage of the TRIF protein, an adaptor protein for TLR3 signaling [42], [45], we observed that MDA5, not RIG-I, was degraded in cells that had been infected with the MP4, the TW2231/98, and the BrCr strains of EV71, and that the MDA5 cleavage was dependent on the caspase activation upon EV71 infection, in a manner similar to that observed in poliovirus infection [40].

In the cytoplasm of cells, the RNA helicases, RIG-I and MDA5, play a key role in the recognition of PAMPs that are generated following RNA virus infection. RIG-I has been implicated in the recognition of RNA with free 5′-triphosphate ends or intermediate length of synthetic poly(I:C), [13], [26]–[28], [30] whereas the agonists of MDA5 protein may be poly(I:C) with long length, long dsRNA derived from positive-stranded RNA virus replication, and highly-ordered RNA structures containing ssRNA and dsRNA [29], [33]. Similar to other picornaviruses, the positive-stranded RNA genome of EV71 is covalently linked to the VPg protein at the 5′terminus. Therefore, the viral genomic RNA may not be recognized by the RIG-I protein. In contrast, by using MDA5^−/−^ knockout mice, several studies have indicated that MDA5 signaling is essential for type-I IFN induction by picornaviruses, such as encephalomyocarditis virus (EMCV), Theiler murine encephalomyelitis virus (TMEV), poliovirus, and coxsackie B virus [14], [34], [37], [46].

In our study, the EV71 viral RNA, which was extracted from the supernatant of EV71-infected Vero cells, was directly delivered to host cells by RNA transfection. Using this procedure, in contrast to natural virus infection, IRF3 phosphorylation and IFN-β expression were activated. Unlike poly(I:C) treatment, this approach provides a system to investigate the regulation of type-I IFN expression in response to the agonist from EV71. Nevertheless, though the purified EV71 viral RNA was shown to primarily consist of positive-stranded EV71 genomic RNA, we cannot exclude absolutely the presence of replication intermediate dsRNA, or cellular RNA contaminants in the RNA preparation. Because we assayed IRF3 activation and IFN-β mRNA expression before large amount of viral protein was expressed, the triggering signal for the response could be the result of the originally transfected viral RNA. However, we do not rule out the possibility that the replication intermediate dsRNA from the second cycle of replication has been generated and involved in the activation of IRF3. To further investigate whether EV71 viral genomic RNA could be the PAMP that is responsible for the MDA5-mediated innate response, in vitro-transcribed viral RNA, that was treated with alkaline phosphatase to remove 5′ phosphates, was used as an agonist for stimulating the expression of IFN-β mRNA (Figure S7). Although this approach can be applied to map the regions that stimulate innate immunity, the efficiency of 5′ phosphate removal may affect the selection of the signal activation pathway. Nevertheless, the properties of EV71 viral RNA that are recognized by MDA5 remain unclear, and should be further studied. Moreover, host responses on viral RNA-transfected cells may be different from natural EV71 infection. The gene expression profiles of host cells should be further analyzed upon EV71 infection to evaluate the innate responses of the infected cells. Since a mouse model has been established using a mouse-adapted strain of the EV71 virus [47], investigations of RIG-I like receptor knockouts, such as MDA5^−/−^ and RIG-I^−/−^, are warranted.

In addition to RIG-I and MDA5, LGP2 is an inducible cytosolic RIG-I like receptor that also contains the DExD/H RNA helicase and the C-terminal repressor domains. Because LGP2 does not contain CARD domains and cannot therefore transduce the activation of downstream molecules through CARD-CARD interactions, LGP2 may function as a negative regulator of type-I IFN activity [23], [24], [48]. However, a recent study showed that LGP2 is required in RIG-I and MDA5 pathways for IFN-β expression during EMCV infection [25]. Although the role of LGP2 in triggering innate immunity remains unclear, studies have shown that the ligand recognized by LGP2 may be blunt-ended dsRNA [49]. Nevertheless, the role of LGP2 in EV71 RNA-mediated IRF3 activation and IFN-β induction requires further investigation.

Many RNA viruses have evolved mechanisms to evade host innate immunity. EV71 inhibits cap-dependent protein translation in infected cells through eIF4G cleavage by its 2A^pro^. Through this mechanism, EV71 may strongly suppress protein synthesis to diminish the host immune response. We found that EV71 infection suppresses total IRF3 expression at the late stage of infection. Under these conditions, poly(I:C) barely stimulated IRF3 activation and IFN-β expression in EV71 infected cells (Figure S8). Nevertheless, previous studies have shown that EV71 infection inhibits specific pathways in the innate response. For example, the EV71 3C protein can disrupt signal activation by blocking RIG-I and degrading TRIF [42], [45]. We further demonstrated that MDA5 is cleaved during EV71 infection, which might partly contribute to the inhibition of IFN-β expression. However, by our viral RNA transfection approach, although EV71 infection caused the degradation of the viral RNA-induced MDA5 (Figure 6), the infection could also provide more EV71 RNA that may stimulate MDA5-mediated IRF3 activation and IFN-β expression. Therefore, other approach is required to determine the effect of MDA5 cleavage on IFN-β production in response to EV71 infection. Furthermore, even though we could detect MDA5 cleavage after 6 h upon EV71 infection, the reasons why IRF3 was not activated in the early time of EV71 infection and why the basal level of endogenous MDA5 did not respond to EV71 infection remain unclear. Although the broad spectrum caspase inhibitor could block MDA5 cleavage and enhance IFN-β production in EV71-infected MDA5 transfectant, we could not rule out that other events generated by the caspase inhibitor could also indirectly change type-I IFN production, such as the increase of cell survival. Thus, elucidating the repression of IFN-β expression during EV71 infection requires further investigation. Because EV71 2A^pro^ potently inhibits host-cap-dependent translation and triggers apoptotic processes [7], it may also be involved in MDA5 degradation, which might contribute to inhibition of IFN-β production. Accordingly, multiple mechanisms are likely responsible for the inhibition of IRF3 activation and IFN-β expression in EV71 infected cells..

## Supporting Information

Figure S1 **MDA5 mediates IRF3 activation in the presence of EV71 RNA in RD cells.** RD cells were transfected with siRNA against MDA5 or scrambled siRNA for 6 h, and cells were subsequently transfected with EV71 RNA for 24 h. (A) Cell extracts were analyzed for MDA5 protein expression by immunoblotting using an anti-MDA5 antibody. (B) Total RNA was isolated from the transfected RD cells, and relative amount of the IFN-β mRNA was measured using real-time RT-PCR.
(TIFF)

Figure S2 **RIG-I siRNA was sufficient to decrease in vitro-transcribed RNA-induced IFN-β expression.** An empty pcDNA3 plasmid was digested with *Eco*RI. RNA was transcribed from the linearized plasmid by MEGAscript in vitro transcription kit (Ambion, USA) with T7 polymerase. The synthesized RNA was purified by RNeasy mini kit (Qiagen, Germany) (A) HeLa cells were transfected with scrambled siRNA or siRNA against RIG-I for 24 h, followed by transfection with 3 µg of the in vitro-transcribed RNA for 20 h. Cell extracts and total RNA were collected from the transfected HeLa cells. The expression of RIG-I, MDA5, phosphorylated IRF3, total IRF3, and β-actin was detected by immunoblotting. (B) Relative amount of IFN-β mRNA in RIG-I knockdown cells was measured by real-time RT-PCR.
(TIF)

Figure S3 **Parallel comparison for MDA5 and RIG-I on IFN-β gene activation upon EV71 infection.** HeLa cells were transfected with an empty plasmid or a plasmid expressing the FLAG-MDA5 or FLAG-RIG-I protein for 38 h. The transfected cells were subsequently infected with the MP4 strain of the EV71 virus at 2 MOI. At 9 h post-infection, cell extracts were analyzed by immunoblotting using anti-FLAG M2, anti-MDA5, anti-RIG-I, anti-3C, and anti-β-actin antibodies. (B) Real-time RT-PCR was performed to measure the relative amount of IFN-β mRNA expression.
(TIFF)

Figure S4 **EV71 induces the cleavage of endogenous MDA5 protein.** HeLa cells were transfected with 1 µg of EV71 RNA. At 14 h post-transfection, the cells were infected with the MP4 strain of the EV7 virus at 2 MOI. At 3 and 6 h post-infection, cell extracts and total RNA were collected for analyzing. (A) Cell extracts were analyzed by immunoblotting using anti-MDA5, anti-EV71, anti-β-actin, and anti-PARP antibodies. (B) Real-time RT-PCR was performed to measure the relative amount of IFN-β mRNA expression.
(TIF)

Figure S5 **Overexpressed MDA5, but not RIG-I, is degraded during EV71 infection.** (A) HeLa cells were transfected with an empty plasmid or a plasmid expressing the FLAG-MDA5 or FLAG-RIG-I proteins for 24 h. The transfected cells were subsequently infected with the MP4 strain of the EV71 virus at 2 MOI. At 9 h post-infection, cell extracts were analyzed by immunoblotting using anti-FLAG M2, anti-3C, and anti-β-actin antibodies. (B) The extracts from the FLAG-MDA5 transfected cells were also analyzed by immunoblotting using an anti-MDA5 antibody. The asterisk indicates the putative cleavage product of the MDA5 protein.
(TIF)

Figure S6 **Examining the effect of caspase-dependent MDA5 cleavage on IFN-β mRNA production upon EV71 infection.** HeLa cells were transfected with a plasmid expressing the FLAG-MDA5 for 22 h The transfected cells were subsequently infected with EV71/MP4 strain in presence or absence of a broad spectrum caspase inhibitor, Q-VD-OPH, at final concentration of 20 nM. Cell extracts and total RNA were collected at 12 h post-infection. The cell extracts were assayed by immunoblotting using anti-MDA5, anti-PARP, anti-V71 3C, and anti-β-actin antibodies (A). Relative amount of IFN-β mRNA in the cells was measured by real-time RT-PCR (B).
(TIF)

Figure S7 **In vitro-transcribed EV71 viral RNA stimulates IFN-β mRNA production.** A plasmid which contains full-length cDNA of EV71/4643 strain in pCR-XL-TOPO vector (kindly provided by Dr. Jen-Ren Wang) was digested with M*lu*I. EV71 viral RNA was transcribed from the linearized plasmid by MEGAscript in vitro transcription kit (Ambion, USA) with T7 polymerase. The synthesized RNA was purified by RNeasy mini kit (Qiagen, Germany) and treated with FastAP alkaline phosphtase (Fermentas, USA) for 30 min at 37°C to remove 5′-triphosphates. After inactivating the enzyme activity at 75°C for 5 min, the dephosphorylated RNA was extracted by phenol-chloroform and precipitated by isopropanol. For the preparation of control RNA, a pcDNA3 empty plasmid was linearized by NotI and performed the in vitro transcription and RNA purification, as described above. HeLa cells were transfected with 2 µg of control or dephosphorylated in vitro-transcribed EV71 RNA. At 24 h post-transfection, total RNA was isolated and analyzed by real-time PCR for measuring relative amount of IFN-β mRNA expression. IVT, in vitro transcription.
(TIF)

Figure S8 **Total IRF3 expression is reduced during EV71 infection.** HeLa cells were infected with the MP4 strain of the EV71 virus. At 4 h post-infection, the cells were transfected with 2 µg of poly(I:C) or transfection reagent alone. At 16 h post-transfection, cell extracts were analyzed by immunoblotting using anti-phospho-IRF3, anti-total IRF3, anti-EV71 3C, and anti-β-actin antibodies.
(TIF)
