# Supplementary figures and images for: MDA5 Plays a Crucial Role in Enterovirus 71 RNA-Mediated IRF3 Activation
Source: PLoS One. 2013 May 1;8(5):e63431. doi: 10.1371/journal.pone.0063431 (PMC3641126; doi:10.1371/journal.pone.0063431)

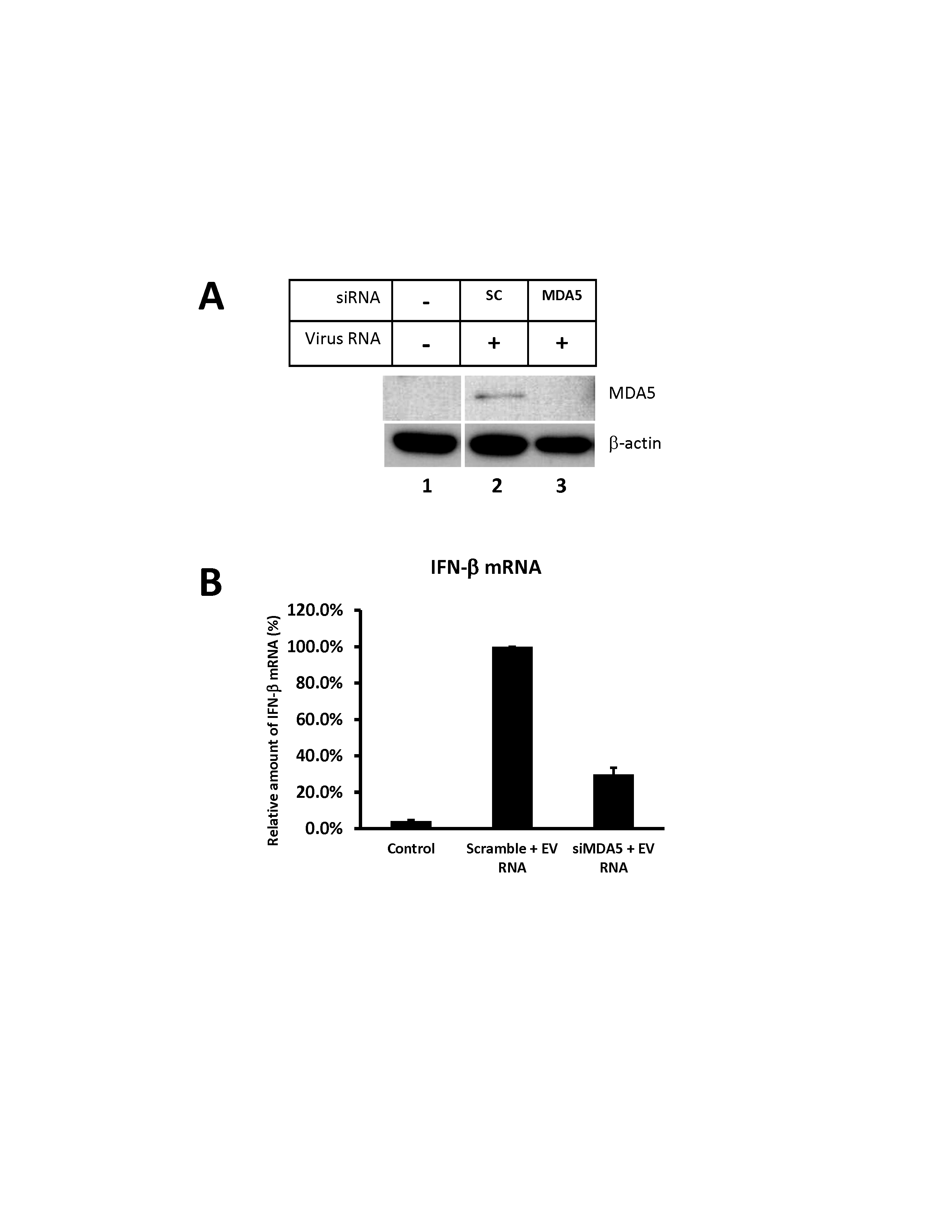

Supplement: Figure S1 — MDA5 mediates IRF3 activation in the presence of EV71 RNA in RD cells. RD cells were transfected with siRNA against MDA5 or scrambled siRNA for 6 h, and cells were subsequently transfected with EV71 RNA for 24 h. (A) Cell extracts were analyzed for MDA5 protein expression by immunoblotting using an anti-MDA5 antibody. (B) Total RNA was isolated from the transfected RD cells, and relative amount of the IFN-β mRNA was measured using real-time RT-PCR. (TIFF) [file pone.0063431.s001.tiff]

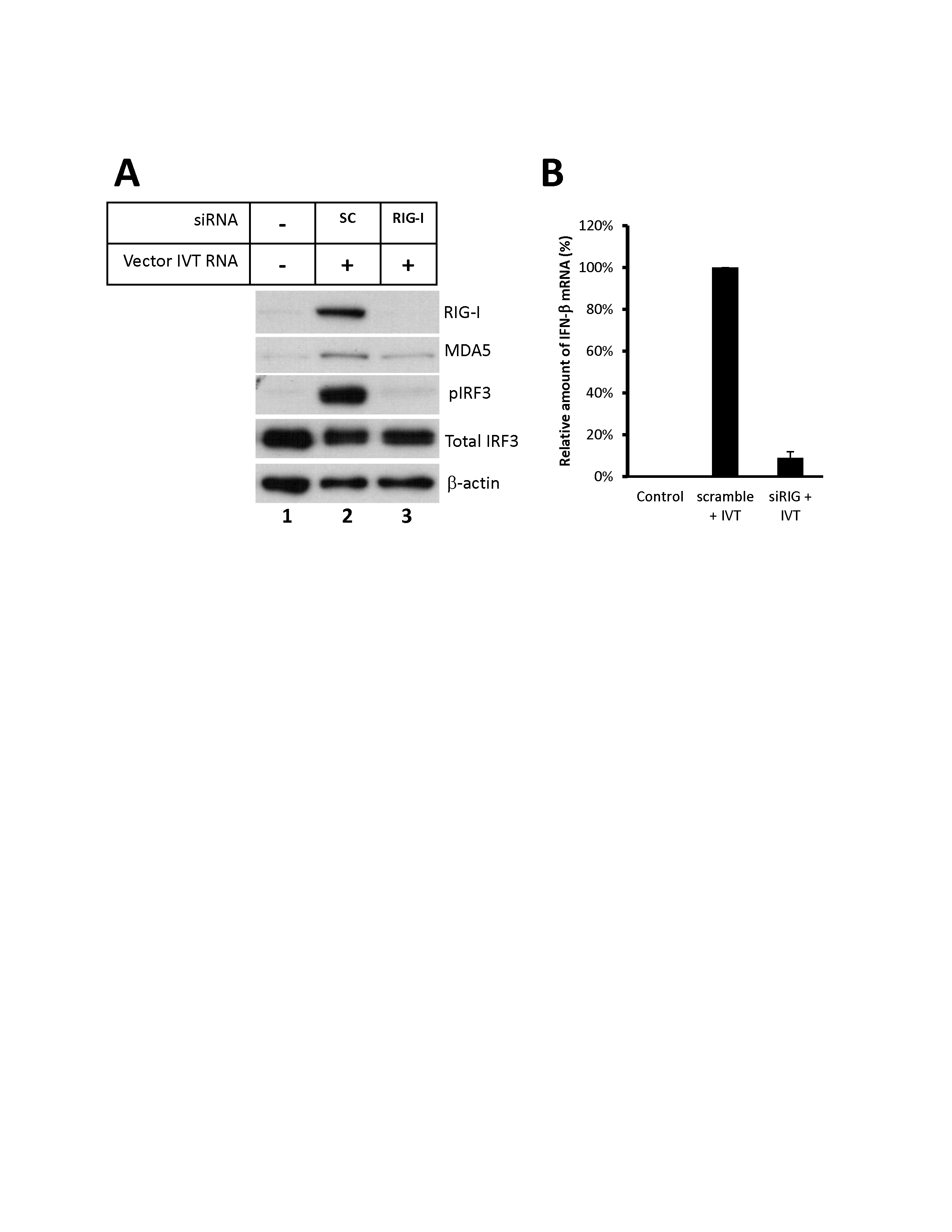

Supplement: Figure S2 — RIG-I siRNA was sufficient to decrease in vitro-transcribed RNA-induced IFN-β expression. An empty pcDNA3 plasmid was digested with EcoRI. RNA was transcribed from the linearized plasmid by MEGAscript in vitro transcription kit (Ambion, USA) with T7 polymerase. The synthesized RNA was purified by RNeasy mini kit (Qiagen, Germany) (A) HeLa cells were transfected with scrambled siRNA or siRNA against RIG-I for 24 h, followed by transfection with 3 µg of the in vitro-transcribed RNA for 20 h. Cell extracts and total RNA were collected from the transfected HeLa cells. The expression of RIG-I, MDA5, phosphorylated IRF3, total IRF3, and β-actin was detected by immunoblotting. (B) Relative amount of IFN-β mRNA in RIG-I knockdown cells was measured by real-time RT-PCR. (TIF) [file pone.0063431.s002.tif]

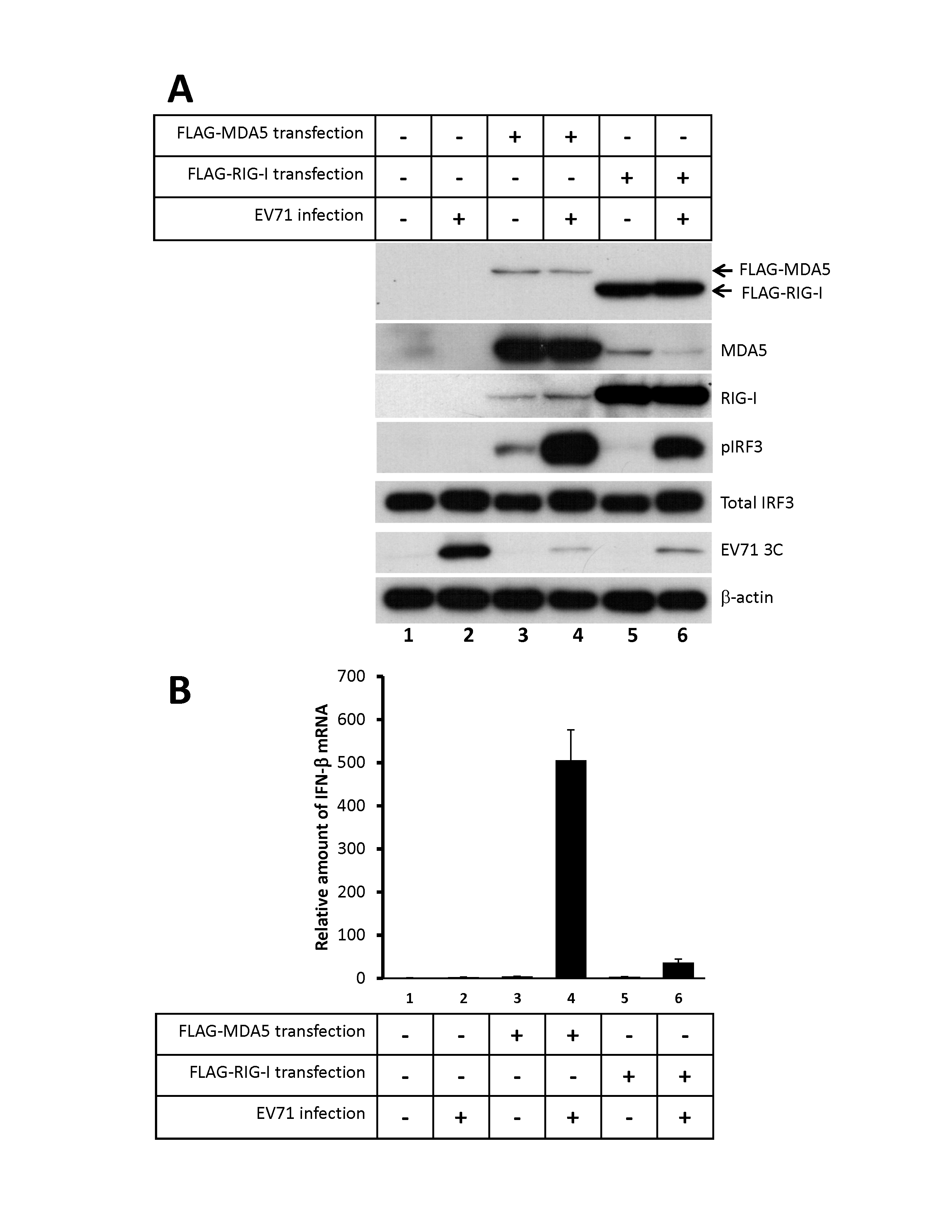

Supplement: Figure S3 — Parallel comparison for MDA5 and RIG-I on IFN-β gene activation upon EV71 infection. HeLa cells were transfected with an empty plasmid or a plasmid expressing the FLAG-MDA5 or FLAG-RIG-I protein for 38 h. The transfected cells were subsequently infected with the MP4 strain of the EV71 virus at 2 MOI. At 9 h post-infection, cell extracts were analyzed by immunoblotting using anti-FLAG M2, anti-MDA5, anti-RIG-I, anti-3C, and anti-β-actin antibodies. (B) Real-time RT-PCR was performed to measure the relative amount of IFN-β mRNA expression. (TIFF) [file pone.0063431.s003.tiff]

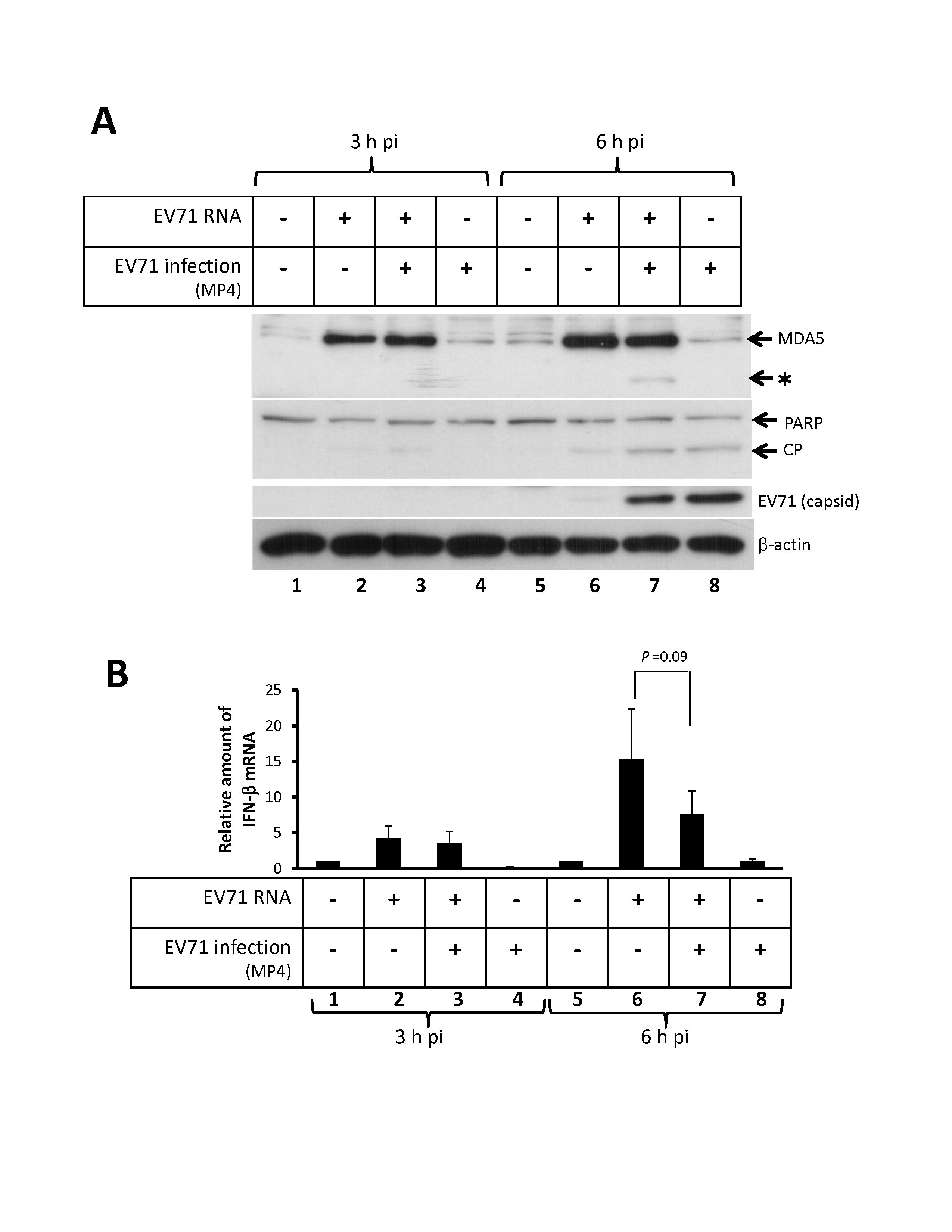

Supplement: Figure S4 — EV71 induces the cleavage of endogenous MDA5 protein. HeLa cells were transfected with 1 µg of EV71 RNA. At 14 h post-transfection, the cells were infected with the MP4 strain of the EV7 virus at 2 MOI. At 3 and 6 h post-infection, cell extracts and total RNA were collected for analyzing. (A) Cell extracts were analyzed by immunoblotting using anti-MDA5, anti-EV71, anti-β-actin, and anti-PARP antibodies. (B) Real-time RT-PCR was performed to measure the relative amount of IFN-β mRNA expression. (TIF) [file pone.0063431.s004.tif]

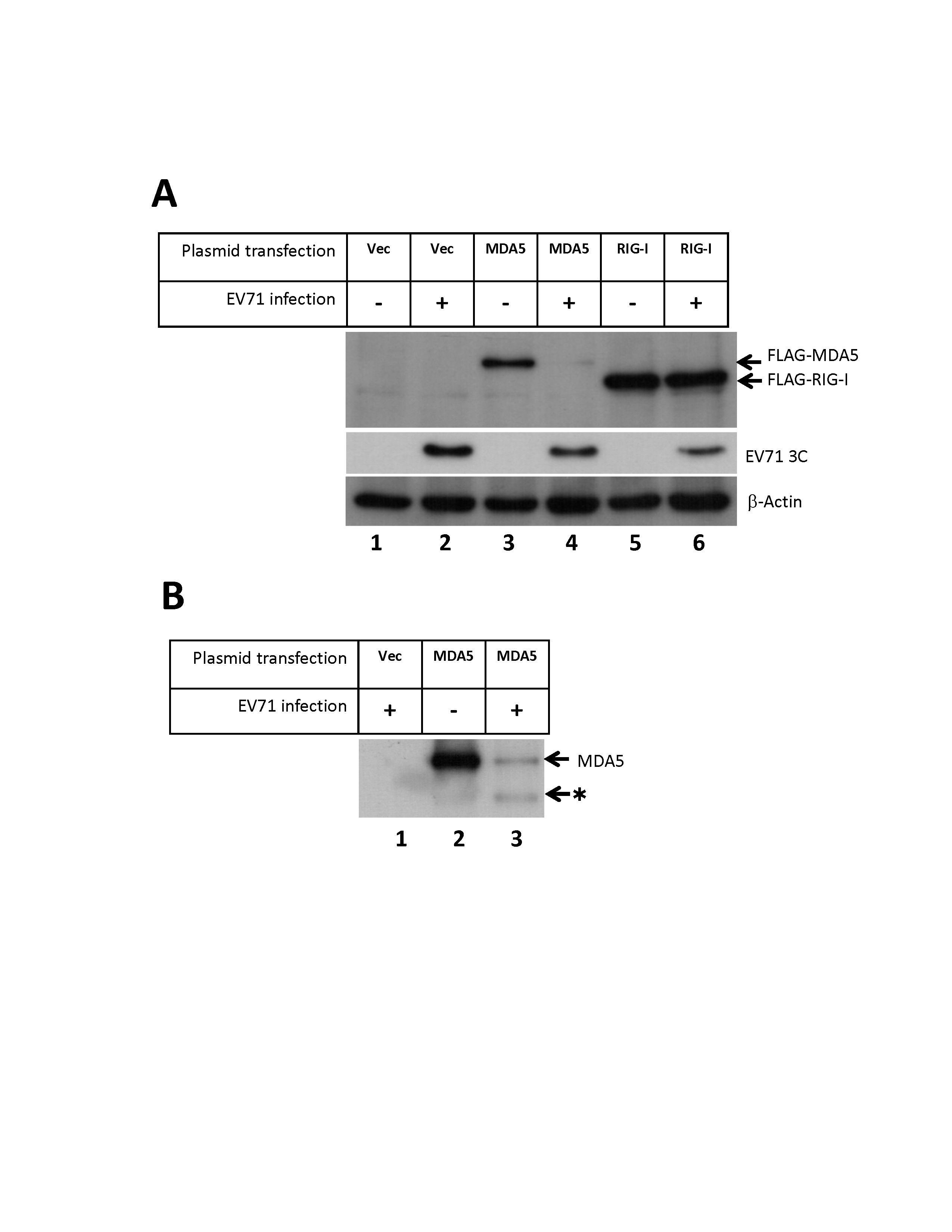

Supplement: Figure S5 — Overexpressed MDA5, but not RIG-I, is degraded during EV71 infection. (A) HeLa cells were transfected with an empty plasmid or a plasmid expressing the FLAG-MDA5 or FLAG-RIG-I proteins for 24 h. The transfected cells were subsequently infected with the MP4 strain of the EV71 virus at 2 MOI. At 9 h post-infection, cell extracts were analyzed by immunoblotting using anti-FLAG M2, anti-3C, and anti-β-actin antibodies. (B) The extracts from the FLAG-MDA5 transfected cells were also analyzed by immunoblotting using an anti-MDA5 antibody. The asterisk indicates the putative cleavage product of the MDA5 protein. (TIF) [file pone.0063431.s005.tif]

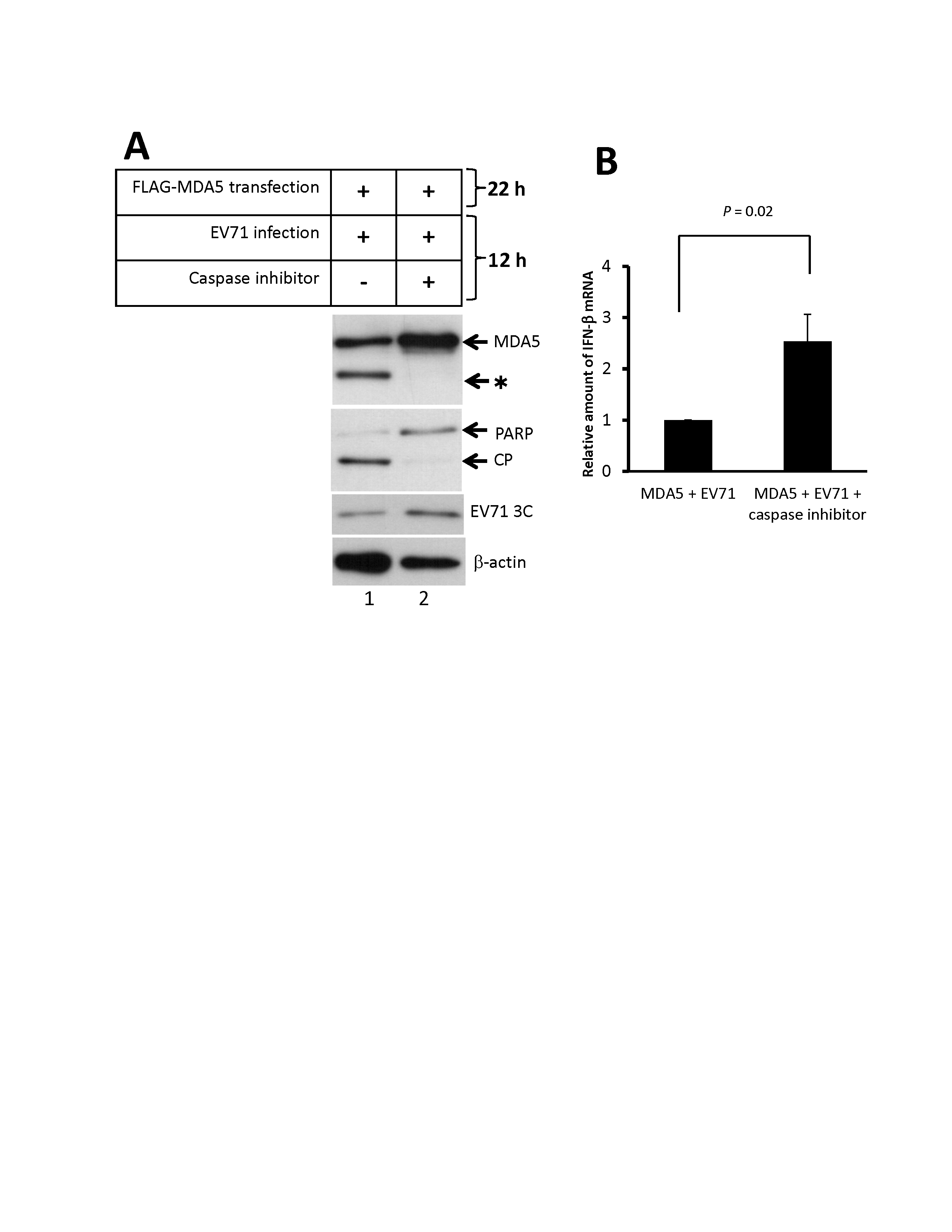

Supplement: Figure S6 — Examining the effect of caspase-dependent MDA5 cleavage on IFN-β mRNA production upon EV71 infection. HeLa cells were transfected with a plasmid expressing the FLAG-MDA5 for 22 h The transfected cells were subsequently infected with EV71/MP4 strain in presence or absence of a broad spectrum caspase inhibitor, Q-VD-OPH, at final concentration of 20 nM. Cell extracts and total RNA were collected at 12 h post-infection. The cell extracts were assayed by immunoblotting using anti-MDA5, anti-PARP, anti-V71 3C, and anti-β-actin antibodies (A). Relative amount of IFN-β mRNA in the cells was measured by real-time RT-PCR (B). (TIF) [file pone.0063431.s006.tif]

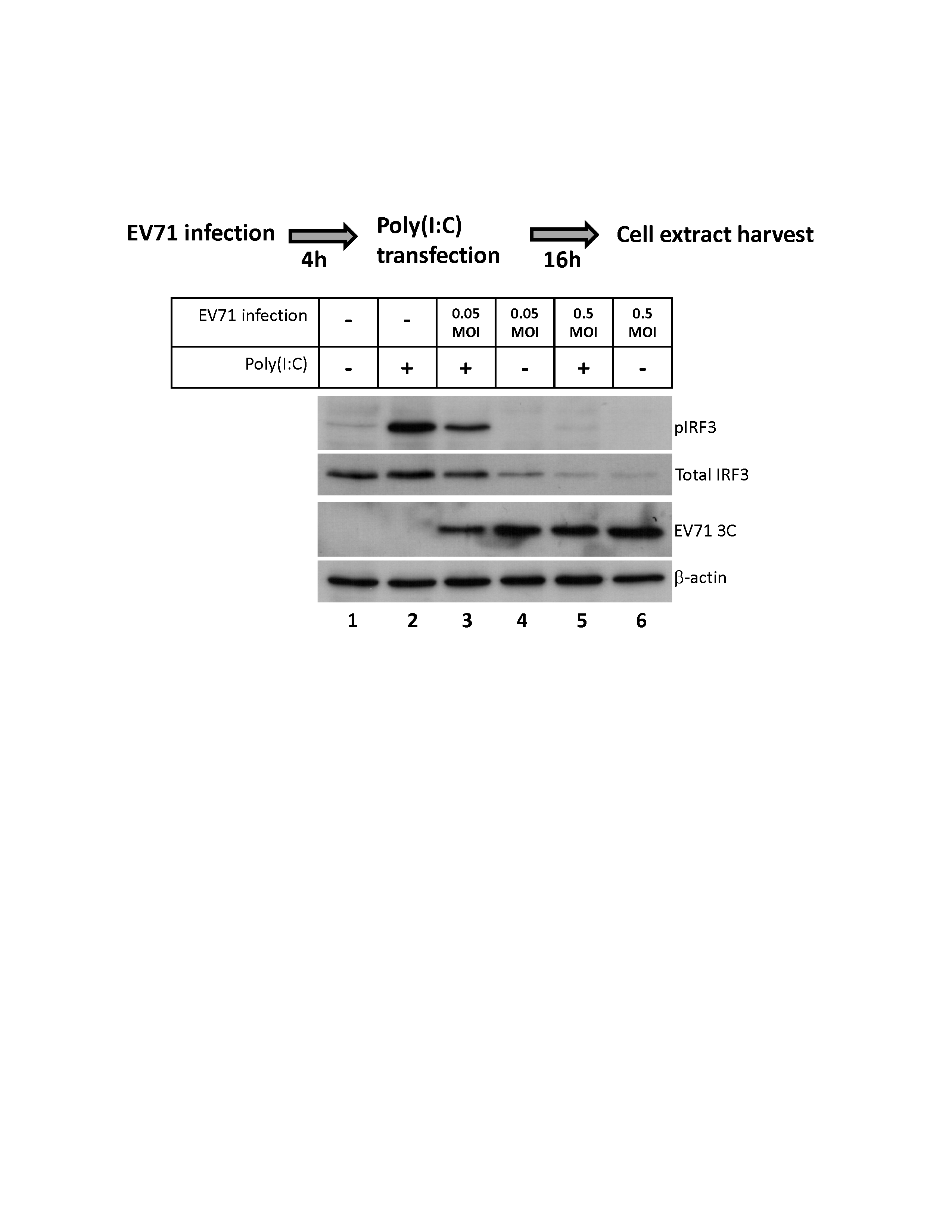

Supplement: Figure S8 — Total IRF3 expression is reduced during EV71 infection. HeLa cells were infected with the MP4 strain of the EV71 virus. At 4 h post-infection, the cells were transfected with 2 µg of poly(I:C) or transfection reagent alone. At 16 h post-transfection, cell extracts were analyzed by immunoblotting using anti-phospho-IRF3, anti-total IRF3, anti-EV71 3C, and anti-β-actin antibodies. (TIF) [file pone.0063431.s008.tif]
